# Optimal design, anti-tumour efficacy and tolerability of anti-CXCR4 antibody drug conjugates

**DOI:** 10.1038/s41598-019-38745-x

**Published:** 2019-02-21

**Authors:** Maria José Costa, Jyothirmayee Kudaravalli, Jing-Tyan Ma, Wei-Hsien Ho, Kathy Delaria, Charles Holz, Angela Stauffer, Allison Given Chunyk, Qing Zong, Eileen Blasi, Bernard Buetow, Thomas-Toan Tran, Kevin Lindquist, Magdalena Dorywalska, Arvind Rajpal, David L. Shelton, Pavel Strop, Shu-Hui Liu

**Affiliations:** 1 Cancer Immunology Discovery, Oncology Research and Development, Worldwide Research and Development, Pfizer Inc., 230 E Grand Ave, South San Francisco, CA 94080 USA; 2 BioMedicine Design, Medicinal Sciences, Worldwide Research and Development, Pfizer Inc., 10646 Science Center Dr, San Diego, CA 92121 USA; 3 Drug Safety Research and Development, Worldwide Research and Development, Pfizer Inc., 10646 Science Center Dr, San Diego, CA 92121 USA; 4 Present Address: Alector, 151, Oyster Point Blvd, suite 300, South San Francisco, CA 94080 USA; 5 Present Address: Grifols Diagnostic Solutions, 6455 Christie Ave B-334C, Emeryville, CA 94608 USA; 6 Present Address: NGM Biopharmaceuticals, Inc, 630 Gateway Blvd, South San Francisco, CA 94080 USA; 7 Present Address: Bristol-Myers Squibb, 700 Bay Rd suite A, Redwood City, CA 94063 USA; 8 Present Address: Multitude Therapeutics, Abmart, Redwood City, CA 94063 USA

## Abstract

Antibody-drug conjugates (ADCs) are promising therapies for haematological cancers. Historically, their therapeutic benefit is due to ADC targeting of lineage-restricted antigens. The C-X-C motif chemokine receptor 4 (CXCR4) is attractive for targeted therapy of haematological cancers, given its expression in multiple tumour types and role in cancer “homing” to bone marrow. However, CXCR4 is also expressed in haematopoietic cells and other normal tissues, raising safety challenges to the development of anti-CXCR4 ADCs for cancer treatment. Here, we designed the first anti-CXCR4 ADC with favourable therapeutic index, effective in xenografts of haematopoietic cancers resistant to standard of care and anti-CXCR4 antibodies. We screened multiple ADC configurations, by varying type of linker-payload, drug-to-antibody ratio (DAR), affinity and Fc format. The optimal ADC bears a non-cleavable linker, auristatin as payload at DAR = 4 and a low affinity antibody with effector–reduced Fc. Contrary to other drugs targeting CXCR4, anti-CXCR4 ADCs effectively eliminated cancer cells as monotherapy, while minimizing leucocytosis. The optimal ADC selectively eliminated CXCR4^+^ cancer cells in solid tumours, but showed limited toxicity to normal CXCR4^+^ tissues, sparing haematopoietic stem cells and progenitors. Our work provides proof-of-concept that through empirical ADC design, it is possible to target proteins with broad normal tissue expression.

## Introduction

The discovery of CXCR4 as a co-receptor for T-tropic HIV-1 variants prompted a wealth of research into its biology and the development of CXCR4 small molecule inhibitors^1^. Besides its function in HIV-1 infection, CXCR4 plays key roles during ontogenesis: chemotaxis of neural and vascular progenitors, migration of haematopoietic precursors from foetal liver to bone marrow and B-lymphocyte and myeloid cell development^2^. As such, global knockouts of CXCR4 and its ligand CXCL12 are embryonic lethal^3–5^. In adult tissues, CXCR4 is expressed in haematopoietic cells, adrenal gland, and kidney tubules^6–8^, whereas CXCL12 is a homeostatic chemokine, being expressed by mesenchymal stromal cells in many tissues^9^. CXCL12/CXCR4 signalling has multiple functions in haematopoietic progenitor cells: maintenance of quiescence, retention in bone marrow and protection from oxidative stress^10–13^. CXCR4 is also required for retention of granulocytic progenitors and neutrophils in the bone marrow^14^. CXCR4 expression is often up-regulated in haematological malignancies^15^, and correlates with therapy resistance and poor prognosis in acute myelogenous leukaemia (AML) and non-Hodgkin lymphoma (NHL)^16–19^. CXCR4^+^ haematological and solid tumour cells co-opt the role of CXCL12/CXCR4 in development and the “homing” of cancer cells to bone marrow is associated with therapy resistance and poor prognosis^20,21^. Among chemokine receptors, CXCR4 is the most widely expressed in solid tumours^22,23^. However, contrary to its endogenous and homogeneous expression in haematological cancers, CXCR4 expression in solid tumour cancer cells is ectopic and heterogeneous, mostly observed in cells displaying tumour-initiating and/or metastatic abilities^23–26^.

Blocking CXCR4 with small molecule (Plerixafor/Mozobil) is approved for CD34^+^ heme progenitors harvest prior to haematopoietic stem cell transplantation in multiple myeloma (MM) and NHL therapy^27^. Targeting CXCR4 is viewed as a promising therapeutic strategy in haematology-oncology indications^28–31^. CXCR4-blocking small molecules or peptides have advanced into clinical trials. However, they often present unfavourable pharmacokinetic profiles, which limit therapeutic benefit and require combination with other therapeutic approaches^30,32,33^. Recently, high affinity CXCR4-blocking antibodies were introduced in the clinic for treatment of haematological cancers^15,34–37^. The therapeutic benefit of CXCR4 blocking approaches is also being tested in solid tumours, thus far with a disappointing outcome^33^.

We aimed to develop an anti-CXCR4 ADC to target haematological cancers refractory to standard of care (SoC) and/or anti-CXCR4 antibodies. ADCs are an appealing drug modality for haematological malignancies, due to lineage-restricted antigen expression, but CXCR4 expression in various adult normal cells raises safety concerns towards anti-CXCR4 ADCs. CXCR4 endocytosis is involved in CXCL12-mediated chemotaxis and CXCR4 cross-linking by antibodies also triggers receptor internalization^35,38,39^. The reported anti-tumour efficacy of a research grade, DAR2, high affinity, anti-CXCR4 ADC demonstrated that a CXCR4:ADC complex can be efficiently internalized^40^. However, this ADC also caused toxicity in normal haematopoietic stem cells and progenitors and it remains unknown whether it presents favourable therapeutic index (TI) in aggressive haematological cancer models^40^. Given the safety concern and our goal to enhance anti-tumour efficacy beyond that of SoC- and CXCR4 antibody-based therapies, we set out to empirically determine the optimal anti-CXCR4 ADC configuration. We found that DAR4 is required in AML models, as well as in therapy-resistant MM xenografts but that a low affinity antibody backbone enhances the TI. Furthermore, the lead anti-CXCR4 ADC demonstrated antineoplastic activity in CXCR4^+^ solid tumour xenograft models. To our knowledge, this is the first report defining the optimal properties of anti-CXCR4 ADCs to maximize their therapeutic potential.

## Results

### Development and characterization of anti-CXCR4 antibodies

**Figure 1:**
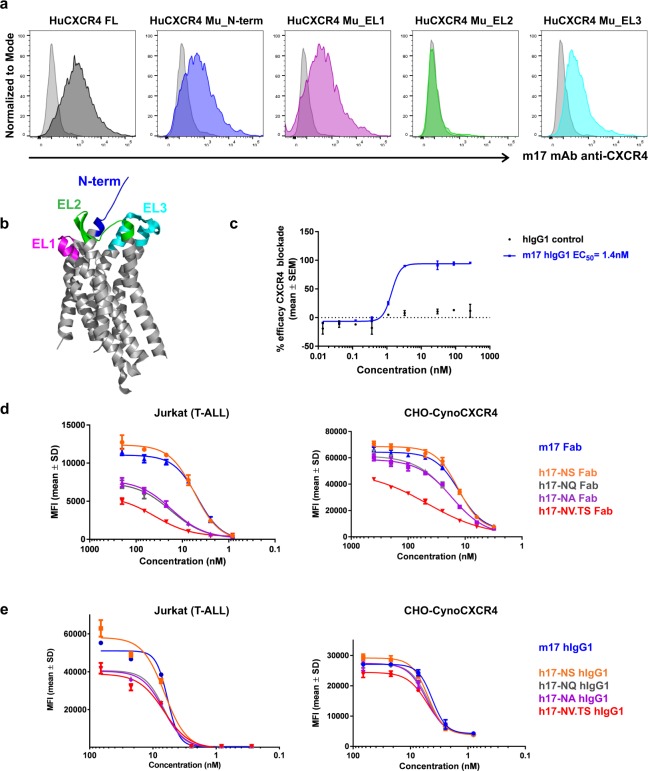
Characterization of antibody backbones for anti-CXCR4 ADCs. (**a**,**d**,**e**), Cell binding assays (flow cytometry). (**a**) CHO cells expressing full length human CXCR4 (HuCXCR4 FL) or chimeric human CXCR4 bearing one of four alternate murine CXCR4 (HuCXCR4 Mu) extracellular domains - N-terminus (N-term) and extracellular loops (EL) 1–3 - screened for binding with m17 mAb. Light grey histogram = Secondary Ab only. Coloured histograms = m17 mAb + secondary Ab. Histogram colours correspond to various human CXCR4 extracellular domains replaced by respective mouse CXCR4 sequences, as represented in b. (**b**) Human CXCR4 with extracellular domains highlighted: N-term = N-terminus, EL1 = extracellular loop 1, EL2 = extracellular loop 2, EL3 = extracellular loop 3. (**c**) Bioassay of CXCR4 blockade with m17 mAb and isotype control, in the presence of EC_80_ forskolin and EC_80_ CXCL12, SEM = standard error of the mean. (**d**,**e**) Binding of serial diluted F(ab) (**d**) or IgG (**e**) on human CXCR4++++ cells (Jurkat) and on CHO-cynoCXCR4 cells, SD = standard deviation.

We generated a chimeric, high affinity, anti-CXCR4 antibody, designated as m17, that showed binding on the human NHL Ramos cell line, but not on CHO cells or CHO expressing mouse CXCR4 (Supplementary Fig. 1a,b). To determine the binding region(s) of m17 on CXCR4, we generated chimeric cDNA constructs by swapping each of the human CXCR4 extracellular domains by its murine counterpart. We found that extracellular loop two (EL2), which contains the epitope for other CXCR4 antibodies and is critical for CXCL12 engagement^34,41^, is also involved in m17 binding (Fig. 1a,b). Accordingly, m17 blocked CXCL12-mediated activation of G_i_ proteins in CXCR4-expressing cells (Fig. 1c). Humanization of m17 produced an initial antibody sequence (h17) that was affinity matured, and optimized, into multiple sequence variants. F(ab) of the top four cell binding variants were selected for further characterization. None of the h17 variants were cross-reactive to mouse CXCR4 (Supplementary Fig. 1b) but they were cross-reactive to cynomolgus (cyno) CXCR4 with same binding ranking as to human CXCR4 (Fig. 1d). Taking EC_50_ values of F(ab) binding on cells expressing high levels of CXCR4 (CXCR4^High^), together with SPR measurements of dissociation rate constants (Table 1 and Supplementary Table 1, respectively), we ranked the binding of the four h17 variants as “high” (h17-NS), “medium” (h17-NQ and h17-NA) and “low” (h17-NV.TS). Bivalent IgG of all h17 variants showed high and similar binding on human CXCR4^High^ cells (Fig. 1e). However, binding of h17 IgG variants diverged on CXCR4^Low^ cells, with the same ranking as that of (monovalent) h17 F(ab)s binding on CXCR4^High^ cells (Supplementary Fig. 1c). Thus, the differentiated binding behaviour of h17 IgG variants on cells with different CXCR4 density levels is likely due to avidity. In an analogous manner to their precursor m17, all h17 variants blocked CXCL12-mediated activation of CXCR4 (Supplementary Fig. 1d).

**Table 1:** Properties of anti-CXCR4 and non-target control ADCs researched in this study.

ADC designation	mAb (hIgG1)	F(ab) EC_50_ Jurkat cells (nM)	F(ab) Max MFI Jurkat cells	Location of conjugation site/tag	Type of linker	Linker-payload	DAR	Fc-mediated effector function
513	m17	4.7	11461	CH1 (tag C), CH2 (N297A mutation, site G)	Non-cleavable	AmPEG6C2-Aur0131	4	Reduced
518	m17	4.7	11461	CH2 (N297Q mutation, sites G, H)	Cleavable	AcLys-VC-PABC-0101	4	Reduced
510	m17	4.7	11461	LC C-term (tag F)	Non-cleavable	AmPEG6C2-Aur0131	2	Active
519	m17	4.7	11461	LC C-term (tag F)	Cleavable	AcLys-VC-PABC-0101	2	Active
381	m17	4.7	11461	HC C-term (tag D)	Cleavable	AcLys-VC-PABC-0101	2	Active
553	h17-NA	17.1	7471	LC C-term (tag F)	Cleavable	AcLys-VC-PABC-0101	2	Active
554	h17-NS	5.5	12747	LC C-term (tag F)	Cleavable	AcLys-VC-PABC-0101	2	Active
555	h17-NQ	17.7	7212	LC C-term (tag F)	Cleavable	AcLys-VC-PABC-0101	2	Active
556	h17-NV.TS	39.7	5015	LC C-term (tag F)	Cleavable	AcLys-VC-PABC-0101	2	Active
669	h17-NQ	17.7	7212	LC C-term (tag F)	Non-cleavable	AmPEG6C2-Aur0131	2	Active
670	h17-NV.TS	39.7	5012	LC C-term (tag F)	Non-cleavable	AmPEG6C2-Aur0131	2	Active
671	h17-NA	17.1	7471	LC C-term (tag F)	Non-cleavable	AmPEG6C2-Aur0131	2	Active
672	h17-NS	5.5	12747	LC C-term (tag F)	Non-cleavable	AmPEG6C2-Aur0131	2	Active
711	h17-NA	17.1	7471	CH1 (tag C), CH2 (N297A mutation, site G)	Non-cleavable	AmPEG6C2-Aur0131	4	Reduced
712	h17-NA	17.1	7471	LC C-term (tag F), CH1 (tag C)	Non-cleavable	AmPEG6C2-Aur0131	4	Active
713	h17-NV.TS	39.7	5015	CH1 (tag C), CH2 (N297A mutation, site G)	Non-cleavable	AmPEG6C2-Aur0131	4	Reduced
714	h17-NV.TS	39.7	5015	LC C-term (tag F), CH1 (tag C)	Non-cleavable	AmPEG6C2-Aur0131	4	Active
217	NNC	N/A	N/A	LC C-term (tag F)	Cleavable	AcLys-VC-PABC-0101	2	Active
675	NNC	N/A	N/A	LC C-term (tag F)	Non-cleavable	AmPEG6C2-Aur0131	2	Active
358	NNC	N/A	N/A	CH2 (N297Q mutation, sites G, H)	Cleavable	AcLys-VC-PABC-0101	4	Reduced
560	NNC	N/A	N/A	CH1 (tag C), CH2 (N297A mutation, site G)	Non-cleavable	AmPEG6C2-Aur0131	4	Reduced
715	NNC	N/A	N/A	LC C-term (tag F), CH1 (tag C)	Non-cleavable	AmPEG6C2-Aur0131	4	Active

**Figure 2:**
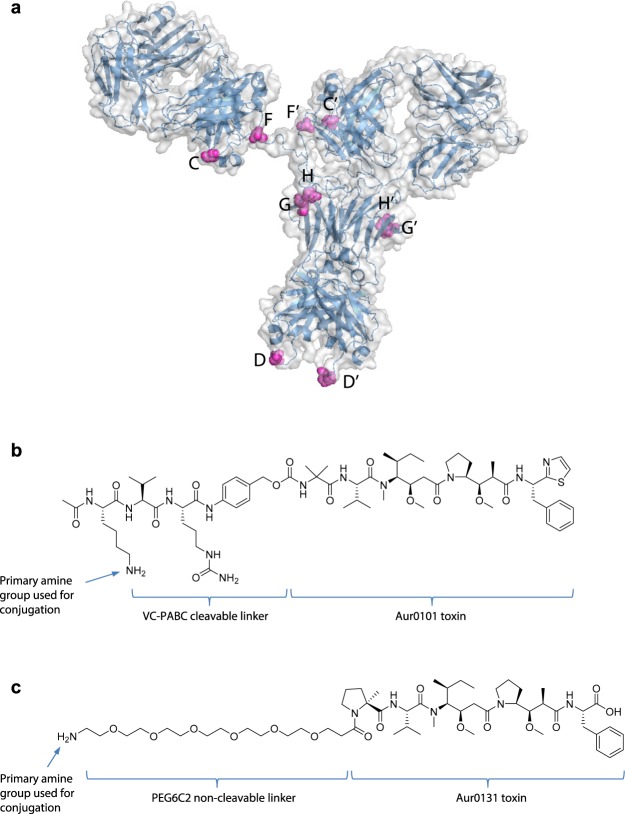
Configuration of the various anti-CXCR4 ADCs investigated in this study. (**a**) Letters indicate location of each of the tested conjugation sites/tags on an antibody (see also Table 1). (**b**) Structure of the cleavable linker payload AcLys-PABC-VC-Aur0101. (**c**) Structure of the non-cleavable linker-payload AmPEG6C2-Aur0131.

See also Fig. 2 and Supplementary Table 1 for further details on ADC configuration and properties.

### Selection of linker-payload and DAR

Besides haematopoietic progenitors, CXCR4 is expressed in leukocytes and other normal adult quiescent tissues (kidney tubular epithelium and adrenal gland)^6,8,42,43^. We reasoned that a payload class whose mechanism of action (MoA) involves blocking cell division would enhance the TI of an anti-CXCR4 ADC. We screened the cytotoxicity of various payloads on tumour cells (Ramos and OPM2) as free drugs (or in a membrane-permeable form) using an *in vitro* assay. After a 48-hour incubation, among the payload classes tested, the three species of the tubulin polymerization inhibitor auristatin caused significant loss of viability on tumour cells (Supplementary Fig. 2a,b). We then tested the cytotoxicity of auristatins on peripheral blood mononuclear cells (PBMCs, an example of CXCR4^+^ quiescent cells). As hypothesized, auristatins did not cause cytotoxicity on PBMCs, at least within the 48-hour incubation (Supplementary Fig. 2c,d). Though we found expression of the multi-drug resistance gene P-glycoprotein 1/ABCB1 in normal PBMCs, its levels were equal or lower to those of tumour cell lines (Supplementary Fig. 2e). Thus, it is unlikely that decreased cytotoxicity of auristatins to normal PBMCs as compared to cancer cells is due to higher P-glycoprotein 1-mediated payload extrusion in PBMCs. We therefore selected auristatins as a payload type suitable for anti-CXCR4 ADCs.

**Figure 3:**
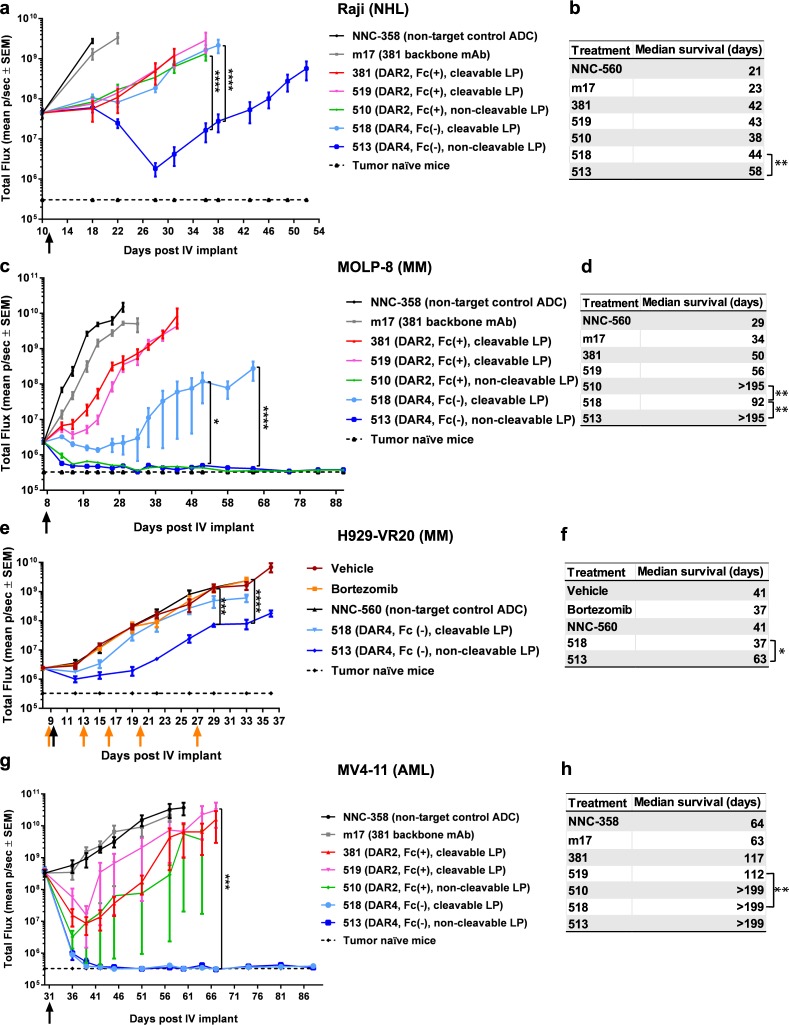
*In vivo* screening of optimal linker-payload and DAR. Orthotopic xenografts of haematological cancers were single dosed (3 mg/kg) with m17-derived ADCs, non-targeted ADC control (NNC) or unconjugated m17 backbone for ADC 381, N = 5/group, black arrows = ADC dosing day. (**a**,**c**,**e**,**g**) Kinetics of whole body tumour burden, LP = linker-payload, Fc(+) = active Fc-mediated effector function, Fc(−) = reduced Fc-mediated effector function, SEM = standard error of the mean, (**a**)****P < 0.0001, (**c**) *P = 0.02 and ****P < 0.0001 (**e**) ***P = 0.0008, ****P < 0.0001, (**g**) ***P = 0.004, two-way ANOVA with Tukey’s multiple comparisons test. (**b**,**d**,**f**,**h**) Kaplan-Meier analysis of median survival, (**b**) **P = 0.003, (**d**) **P = 0.004, (**f**) *P = 0.02 (**h**) **P = 0.004. (**a**) Raji xenografts were treated at high tumour burden to assess anti-tumour activity beyond that of unconjugated anti-CXCR4 hIgG1 control. (**e**) Yellow arrows = dosing days in bortezomib (0.8 mg/kg/dose, N = 5 mice) and vehicle control (N = 4 mice) treated groups.

We compared the cytotoxicity among potential auristatin-based ADC configurations *in vitro* using m17 antibody backbone as an affinity anchor (Table 1 and Fig. 2). The conjugation sites and linkers tested herein were selected based on previous studies indicating their favourable impact on ADC stability and efficacy^44–47^. We used a panel of cell lines derived from NHL, acute lymphocytic leukaemia (ALL), MM and AML, because these are the haematological cancer types most likely to present as SoC-refractory/relapsed disease. We estimated CXCR4 density in plasma membrane and found that CXCR4 expression levels rank from high to medium in NHL and ALL, and from medium to low in MM and AML. CXCR4 expression was higher in tumour cell lines than in normal bone marrow haematopoietic cells (Supplementary Table 2). In most cell lines tested in an *in vitro* cytotoxicity screen, the non-cleavable linker-payload AmPEG6C2-Aur0131 conjugated at DAR4 on antibody sites C and G (ADC 513) resulted in the most efficacious ADC format (Supplementary Table 3). We then tested *in vivo* the anti-tumour activity of m17-derived ADCs in orthotopic xenografts. Similarly, ADC 513 was the most efficacious configuration in NHL, MM and ALL xenografts (Fig. 3a–f and Supplementary Fig. 3a–f). AmPEG6C2-Aur0131 was efficacious even when conjugated at DAR2 (ADC 510) in MOLP-8 xenografts, a MM model resistant to SoC bortezomib and anti-CXCR4 antibodies, in spite of bearing high CXCR4 surface expression^36^ (Fig. 3c,d, Supplementary Fig. 3g, Supplementary Table 2). In the H929-VR20 MM model, selected *in vitro* as a H929 sub-population resistant to 20 nM bortezomib (Supplementary Fig. 3g), CXCR4 expression is up-regulated (Supplementary Fig. 3h) and ADC 513 caused significant tumour growth inhibition (Fig. 3e,f). In contrast to other tumour types, AML models required DAR4 and there was no significant difference in cytotoxicity between the two linker-payload auristatin modalities (ADC 518 versus ADC 513), either *in vitro* or *in vivo* (Fig. 3g,h and Supplementary Table 3). This result is consistent with higher resistance of AML cells to both auristatin species, as compared to other tumour types (Supplementary Table 3). However, these observations did not correlate with detectable differences in either cell proliferation rates or activity of ABC transporters associated with multi-drug resistance, including P-glycoprotein 1, between AML and other tumour types (Supplementary Fig. 4). In most tested tumour models, the ranking of efficacy for m17-derived ADC formats was consistent *in vitro* and *in vivo*, with ADC 513 bearing the most efficacious configuration. Sensitivity to ADC 513 did not correlate with CXCR4 cell surface density and ADC 513 caused cytotoxicity in all haematological tumour models with detectable CXCR4 surface expression (Supplementary Tables 2–3).

### Mechanism of anti-CXCR4 ADC-mediated cytotoxicity

*In vitro*, ADC 513 blocked CXCR4 activation as efficiently as its unconjugated antibody backbone (Supplementary Fig. 5a). The ability to mobilize CXCR4^+^ cells from bone marrow into peripheral blood (leucocytosis) is a hallmark of CXCR4 blocking agents. Three hours post dose, ADC 513 did not cause leucocytosis of AML blasts in MV4–11 xenografts, whereas detectable tumour mobilization was observed in mice dosed with its respective unconjugated antibody (Supplementary Fig. 5b). Instead, ADC 513 tended to decrease blast counts in the bone marrow (Supplementary Fig. 5c,d). The fast tumour killing correlated with tumour stasis and regression at 24 and 48 hours, respectively, post ADC 513 administration to a parallel mouse cohort (Supplementary Fig. 5e). At 1.5 hours post ADC 513 dose, there was an increase in MV4–11 blasts bearing phosphorylated histone-H3^Ser10^ in bone marrow, suggesting mitotic arrest (Supplementary Fig. 5f). These results correlated with a (non-significant) trend for increased tumour cell death during the same period (Supplementary Fig. 5g). We conclude that ADC 513 blocks CXCR4 signalling and induces cytotoxicity *in vivo* through a classic tubulin polymerization inhibitor (payload-dependent) mechanism, thereby minimizing tumour leucocytosis.

### Determination of optimal antibody sequence and Fc format for anti-CXCR4 ADCs

**Figure 4:**
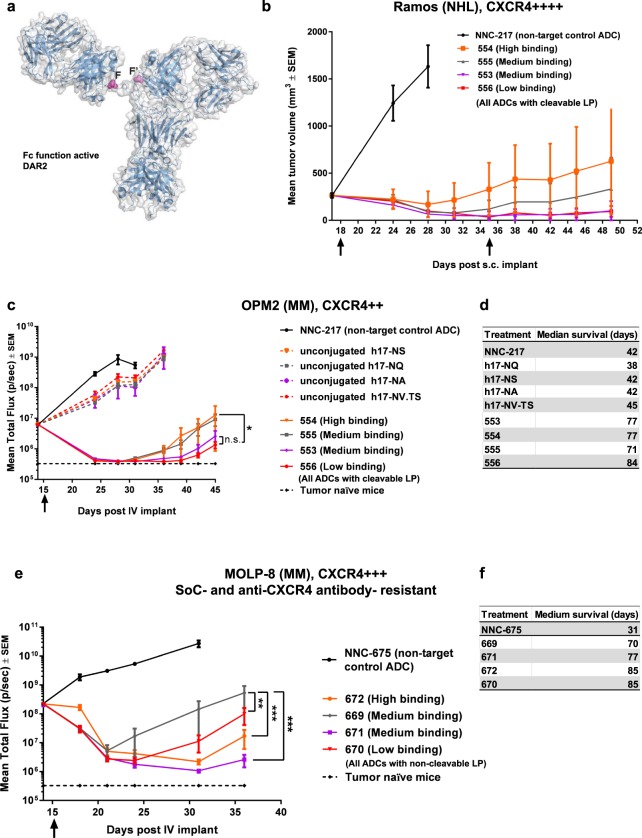
*In vivo* screening of optimal antibody backbone sequence for anti-CXCR4 ADCs. (**a**) Attachment sites of linker-payload (either cleavable or non-cleavable) on constant region of antibody light chains in DAR2 h17-derived ADC variants. (**b**,**c**,**e**) Kinetics of tumour growth, all ADCs dosed at 3 mg/kg (arrows indicate dosing days), LP = linker-payload, N = 5/group, SEM = standard error of the mean, two-way ANOVA with Tukey’s multiple comparisons test (**c**) *P = 0.02, n.s. = non-significant, (**e**) **P = 0.002, ***P = 0.0002, differences between other groups not statistically significant. (**b**) Sub-cutaneous Ramos-derived xenografts. (**c**,**e**) Kinetics of whole-body tumour burden. (**d**,**f**) Kaplan-Meier analysis of median survival: differences in median survival among ADC-treated cohorts did not reach statistical significance, but statistically significant differences between ADCs and unconjugated antibodies were found (P < 0.0001). (**c**,**d**) OPM2 cells were orthotopically implanted. Unconjugated h17 hIgG1 variants were included as controls. (**e**,**f**) MOLP-8 cells (resistant to SoC and anti-CXCR4 antibodies) were implanted orthotopically. AmPEG6C2-Aur0131-derived ADCs were used in this study, as AcLys-VC-PABC-0101 is not efficacious in this model.

To better assess optimal antibody sequence, we tested the anti-tumour efficacy of the four h17 variants (i.e. different binding variants after humanization) with conjugates built at the sub-optimal DAR2 (Fig. 4a). All ADCs harboured active Fc-mediated effector function, to compare ADC efficacy with that of anti-CXCR4 “naked” antibody (hIgG1) approaches. In an *in vitro* cytotoxicity screen, the ADC with the strongest binding (ADC 554) showed the best efficacy (Supplementary Table 4). To screen for optimal antibody sequence *in vivo*, we used the linker-payload type (at DAR2) required for anti-tumour activity in each xenograft model, as determined in the experiments with m17-derived ADCs (see Fig. 3 and Supplementary Fig. 3a–f). Treatments were initiated at high tumour burden to further increase the stringency of the *in vivo* screening. In contrast to the results of the *in vitro* screen, *in vivo*, ADCs of the medium (ADC 553) and low (ADC 556) cell binding variants tended to display the best efficacy in both CXCR4^High^ and CXCR4^Low^ tumour xenografts (Fig. 4b–d). In the SoC- and anti-CXCR4 antibody- resistant model MOLP-8, ADCs of medium (ADC 671) and low (ADC 670) cell binding were as efficacious as the one featuring high cell binding (ADC 672) (Fig. 4e,f). In all tested xenografts, ADCs derived from medium binding h17-NQ antibody (ADCs 555 and 669) displayed the lowest efficacy, but the reason for this observation was not investigated. Though the various ADCs significantly prolonged median survival when compared to control ADC or unconjugated antibodies, differences among the various ADCs did not reach statistical significance (Fig. 4d,f).

We also tested whether medium and low cell binding ADCs 553 and 556 were able to compete with CXCL12 for binding to CXCR4 in CXCR4^Low^ tumours. To address this question, we compared the *in vitro* ADC cytotoxicity to the MM cell line OPM2, without and with CXCL12 at 100 ng/mL, a concentration that potently induces MM cell migration *in vitro*^48^. Both ADC variants displayed enhanced cytotoxicity in the presence of CXCL12. In contrast, CXCL12 levels did not modulate the cytotoxicity of free payload (Supplementary Fig. 6a,b). We then investigated the effects of pathophysiologically relevant CXCL12 levels on ADC cytotoxicity to MM OPM2 cells. CXCL12 concentration is elevated in the bone marrow of MM patients as compared to bone marrow of healthy subjects^48^. We observed that CXCL12 concentration as found in MM-diseased bone marrow (7 ng/mL) also enhanced cytotoxicity of low cell binding ADC 556 to OPM2 cells (Supplementary Fig. 6c). In our orthotopic *in vivo* models, mouse CXCL12 activates human CXCR4^8,12^ and most MM cells are found in the bone marrow. The serum CXCL12 levels in tumour-naïve NSG mice average those of wild type, tumour-naïve Balb/c mice (Supplementary Fig. 6d). This suggests that the influence of CXCL12 on anti-CXCR4 ADC efficacy in a hypothetical immunocompetent host is correctly modelled in NSG xenografts. Altogether, we concluded that the medium and low cell binding antibody backbones, h17-NA and h17-NV.TS, respectively, provide anti-CXCR4 ADCs with adequate anti-tumour activity *in vivo*.

**Figure 5:**
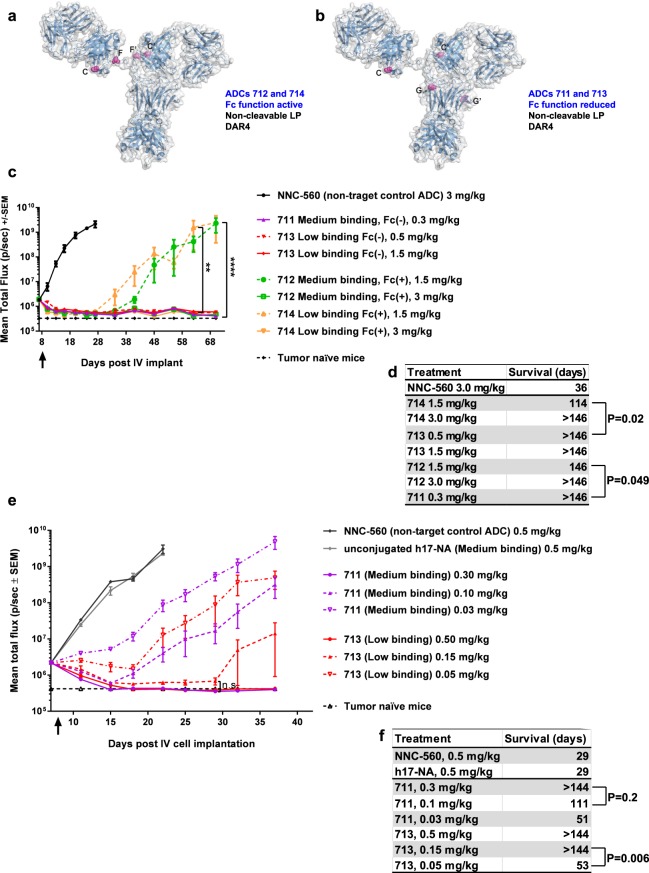
Efficacy studies with MOLP-8-derived xenografts (SoC- and CXCR4 antibody- resistant MM cell line) to select ADC Fc format and determine ADC MED. (**a**) Attachment sites of linker-payload on antibody constant regions of light (tags F, F’) and heavy chains (tags C and C’) in DAR4 ADCs 712 and 714. (**b**) Attachment sites of linker-payload on antibody constant regions of heavy chains (tags C and C’ and sites G and G’) in DAR4 ADCs 711 and 713. The N297A mutation to generate sites G and G’ leads to Fc deglycosylation and reduced effector function. (**a**,**b**) LP = linker payload. (**c**,**d**) Assessment of Fc format. (**e**,**f**) Determination of MED for ADCs 711 and 713. (**c**,**e**) Kinetics of orthotopic tumour growth, with a single ADC dose administered at day 8 (arrow indicates dosing day), N = 5/group, SEM = standard error of the mean, (**c**) **P = 0.006 and ****P < 0.0001, (**e**) n.s. = non-significant, two-way ANOVA with Tukey’s multiple comparisons test. (**c**) Fc (+) = active Fc-mediated effector function, Fc (−) = reduced Fc-mediated effector function. (**d**,**f**) Kaplan-Meier analysis of median survival.

We therefore selected h17-NA and h17-NV.TS sequence variants for conjugation with AmPEG6C2-Aur0131 at DAR4. In addition, we compared the efficacy of h17-NA and h17-NV.TS -derived ADCs with active and reduced Fc-mediated effector function. The N297A mutation allows for linker-payload attachment to site G/G’ on the antibody (Fig. 2a) and causes loss of Fc glycosylation. The interaction Fc:Fcγ receptor, and therefore Fc-mediated effector function, is reduced upon loss of glycosylation at the N297 site. The N297A mutation has been widely used to reduce Fc-mediated effector function in therapeutic antibodies^49,50^. Since we started our studies, another mutation of this site (N297G) has been described which reportedly increases antibody developability^51^. However, the pharmacokinetic profiles of antibodies carrying N297A or N297G mutations are similar in cynomolgus monkeys and we have not tested conjugates with this other sequence^52^. Thus, a total of four AmPEG6C2-Aur0131 DAR4 conjugates were generated and their potency and specific cytotoxicity confirmed *in vitro* (Fig. 5a,b, Table 1 and Supplementary Table 5). The Fc-mediated effector function *in vitro* was comparable between the ADCs and their respective unconjugated antibodies and, as expected, was significantly reduced in molecules bearing the N297A mutation (Supplementary Fig. 7). In MOLP-8-derived xenografts, both medium and low cell binding variants bearing active Fc-mediated effector function (ADCs 712 and 714, respectively) were less efficacious than their counterparts with N297A mutation (ADCs 711 and 713, respectively) (Fig. 5c,d). The lower efficacy of ADCs 712 and 714 correlated with their faster clearance in NSG mice bearing MOLP-8-derived xenografts, as compared to ADCs 711 and 713 (Supplementary Table 6).

### Determination of minimal efficacious dose (MED) of lead anti-CXCR4 ADCs

We proceeded with ADCs 711 and 713 to evaluate TI. Considering that the safety/tolerability assessment is based on repeat dosing once every 3 weeks, we defined MED as the lowest tested single dose causing tumour regression sustained for 3 weeks, in the SoC- and CXCR4 antibody- resistant MOLP-8 xenograft model. We found that the MED of lead ADCs in this model is 0.15 mg/kg for ADC 713 and 0.1 ≤ 0.3 mg/kg for ADC 711 (Fig. 5e,f).

### Tolerability of lead anti-CXCR4 ADCs in mice

**Figure 6:**
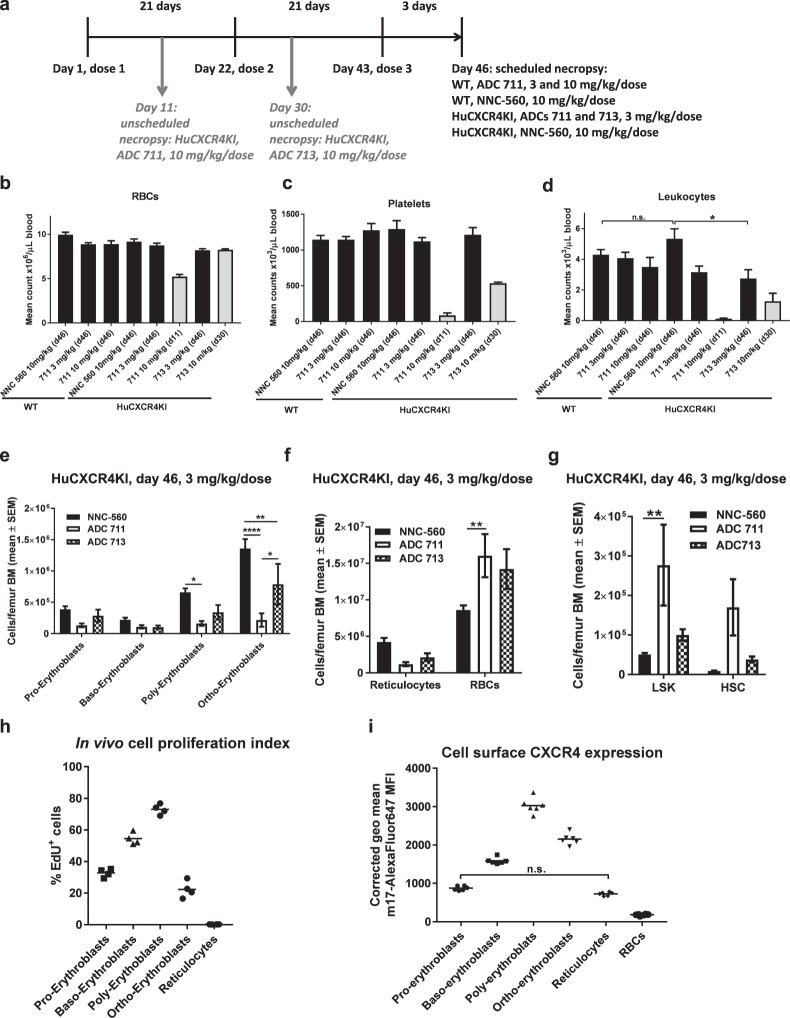
Tolerability of lead anti-CXCR4 ADCs in mice inversely correlates with ADC binding strength and lead ADCs present limited toxicity profile. (**a**) Dosing schedule and time of necropsy during tolerability/safety studies in WT and HuCXCR4KI. Unscheduled necropsies of HuCXCR4KI were due to lack of tolerability to indicated ADC/dose combination. (**b**–**d**) Haematology analysis (necropsy day for each treatment group indicated in parenthesis on *x*-axis), grey bars (N = 3/group, unscheduled necropsies on days 11 and 30), black bars (N = 6/group, scheduled necropsy on day 46). Error bars = standard error of the mean, n.s. = non-significant, *P < 0.02 (One-way ANOVA with Tukey’s multiple comparisons test at day 46). (**e**–**g**) Enumeration of haematopoietic progenitors in femur bone marrow, SEM = standard error of the mean. All analyses from same samples, N = 5/group, two-way ANOVA with Dunnett’s multiple comparisons test (**e**) *P = 0.01, **P = 0.003, ****P = 0.0001. (**f**) **P = 0.009. (**g**) **P = 0.009. (**h**,**i**) Each symbol represents data from an individual mouse, one-way ANOVA with Tukey’s multiple comparisons test. (**h**) Proliferation of erythroid progenitors in bone marrow of HuCXCR4KI in homeostatic conditions, all group comparisons with ****P < 0.0001, except pro-erythroblasts versus ortho-erythroblasts: **P = 0.006. (**i**) Cell surface CXCR4 levels in erythroid progenitors in bone marrow of HuCXCR4KI in homeostatic conditions, all group comparisons with ****P < 0.0001, except where indicated n.s. (non-significant). (**e**,**h**,**i**) baso-erythroblasts = basophilic erythroblasts, poly-erythroblasts = polychromatic erythroblasts, ortho-erythroblasts = orthochromatic erythroblasts.

The tolerability/safety assessment was performed in human CXCR4 knock-in (HuCXCR4KI) mice (Fig. 6a and Supplementary Table 7). In this mouse model, the coding region of mouse CXCR4 gene has been replaced by the human ortholog sequence and the mice develop an overtly normal haematopoietic system^8^. Thus, HuCXCR4KI mice allow the evaluation of normal tissue toxicity of anti-human CXCR4-specific ADCs. As expected, tolerability of anti-CXCR4 ADCs inversely correlated with cell binding: ADC 713 (low binding) was tolerated at 4.5 < 6 mg/kg/dose, whereas ADC 711 (medium binding) was tolerated at 3 < 4.5 mg/kg/dose. In contrast, ADC 711 was tolerated in wild type (WT) mice at ≤10 mg/kg/dose, suggesting minimal off-target toxicity (Supplementary Table 7). Both ADCs appeared stable *in vivo* given the overlapped pharmacokinetic profiles from total antibody and conjugated antibody assays in both NSG bearing MOLP-8 xenografts and HuCXCR4KI mice (Supplementary Table 6). The main toxicity findings in HuCXCR4KI dosed with ADCs 711 and 713 at the non-tolerated 10 mg/kg/dose were in the haematopoietic compartment: thrombocytopenia, erythropenia and leucopenia, due to anti-CXCR4 ADC-related toxicity in bone marrow, spleen and thymus (Fig. 6b–d), whereas no severe toxicity was observed in other CXCR4^+^ adult tissues^6–8,53^. In accordance with tolerability observation, hematopathology findings at 10 mg/kg/dose were more severe with the medium cell binding ADC 711 than with the low cell binding ADC 713 (Fig. 6a–d and Supplementary Fig. 8a–c). At study day 46, 3 mg/kg/dose ADC 713 caused detectable decrease in peripheral blood neutrophils and monocytes. Neither ADC caused detectable decrease in lymphocytes, red blood cells (RBCs) or platelets at the same dose and time point (Fig. 6b–d and Supplementary Fig. 8a–c). However, 10 days after *single* 3 mg/kg dose of ADC 711, RBCs were decreased in the bone marrow (Supplementary Fig. 8d,e). This observation suggests that, in the repeat dosing study at 3 mg/kg/dose, compensatory mechanisms were active in the bone marrow, thus allowing cellularity recovery and thereby, tolerability (see below).

The haematopoietic toxicity profile at 3 mg/kg/dose (study day 46) was more limited than expected, given the broad CXCR4 expression in haematopoietic cells. We hypothesized that the specific toxicity towards the myeloid-erythroid lineage stemmed from differential susceptibility of the various lineage-committed progenitors to anti-CXCR4 ADCs and that a reduction in RBCs, neutrophils and monocytes was a downstream effect. At study day 46, we detected a decrease in granulocyte-monocyte progenitors (GMP) and in specific erythroblast subsets, but not in other lineage-committed precursors from femur bone marrow of anti-CXCR4 ADC-treated HuCXCR4KI (Fig. 6e and Supplementary Fig. 8f). At the same time point, RBCs in bone marrow were increased ≈ 2-fold, suggesting that compensatory mechanisms for RBC recovery were active after first 3 mg/kg dose, as hypothesized (Fig. 6f). Indeed, both haematopoietic stem cells and progenitors (Lin^−^Sca1^+^c-Kit^+^, LSK) and haematopoietic stem cell (HSC) populations were increased in HuCXCR4KI dosed with ADCs 711 and 713 at 3 mg/kg/dose (Fig. 6g). Importantly, CXCR4 ADCs did not cause significant LSK cell mobilization (Supplementary Fig. 8g,h).

Differential surface CXCR4 expression and cell proliferation rates among haematopoietic precursors in homeostatic conditions may explain toxicity of anti-CXCR4 ADCs towards specific progenitors. We found that cell surface CXCR4 density *per se* is not sufficient to predict cytotoxicity of ADCs 711 and 713 in normal adult tissues. Rather, their effect in normal cells is determined by a combined threshold of, at least, high cell surface CXCR4 expression and rapid cell proliferation (Fig. 6h,i and Supplementary Fig. 8i,j). These results are consistent with the MoA of auristatins and further validate our hypothesis that this payload class enhances the TI of anti-CXCR4 ADCs. We postulate that, owing to relatively low CXCR4 expression levels in normal cells, the low cell binding ADC 713 is better tolerated.

Another factor determining ADC cytotoxicity is the ability of cells to internalize receptor:ADC complexes and sort them to lysosome for release of drug payload. To test whether CXCR4:ADC/antibody internalization rates differ between normal and cancer cells, we measured the kinetics of anti-CXCR4 h17-NV.TS (antibody backbone of ADC 713) delivery to lysosome in human cancer cells and normal PBMCs *in vitro*. To this end, h17-NV.TS hIgG1 was pre-bound to F(ab) anti-human IgG labelled with a pH-sensitive fluorophore (whose red fluorescent signal increases at the low pH in lysosome) and added to cells. We found that cancer cells sort anti-CXCR4 antibody to lysosome at higher rate than normal PBMCs (Supplementary Fig. 9a). As a control, we show that an isotype antibody is not internalized (Supplementary Fig. 9b). The high and low cell binding h17 IgG variants exhibited overlapping internalization kinetics in each tested cell type (Supplementary Fig. 9c). We conclude that CXCR4 internalization rate may also contribute to differential cytotoxicity of anti-CXCR4 ADCs to different cell types, including normal versus cancer cells, but it is a cell-specific attribute that is not affected by ADC binding properties.

To estimate the TI of ADCs 711 and 713, we compared the area under the concentration curve (AUC) values at the known tolerated doses in HuCXCR4KI (3 and 4.5 mg/kg, respectively) with those at the known MED in the SoC- and anti-CXCR4 antibody-resistant MOLP-8-derived xenografts (0.3 and 0.15 mg/kg, respectively). The AUC value at 4.5 mg/kg for ADC 713 was scaled proportionately from the 3 mg/kg AUC_504_ values. Based on the pharmacokinetics (Supplementary Table 6), we estimated the TI of ADCs 711 and 713 at ≈ 4.5 and ≈ 8.1, respectively.

### Antineoplastic activity of lead anti-CXCR4 ADC in CXCR4^+^ solid tumours

**Figure 7:**
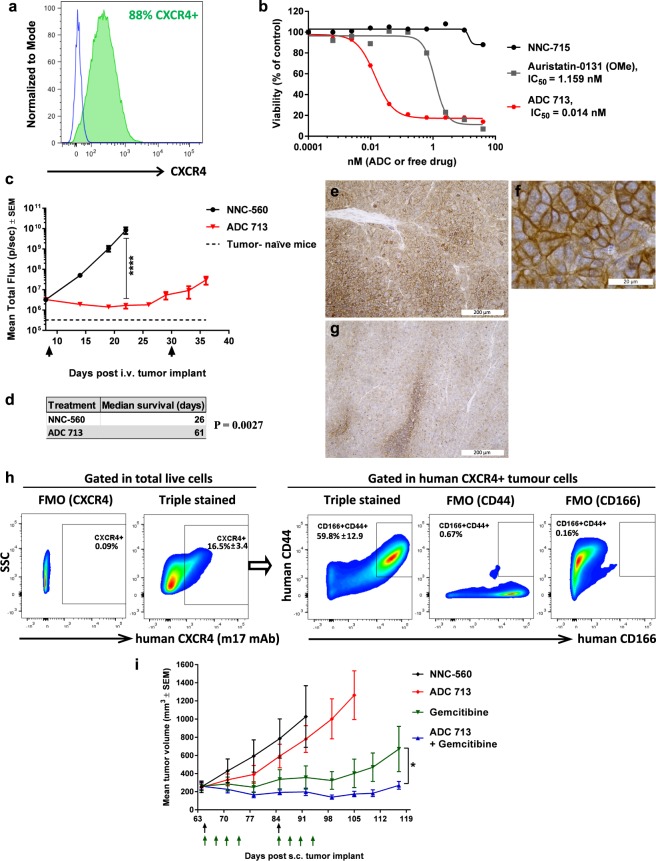
Lead anti-CXCR4 ADC 713 shows antineoplastic activity in CXCR4^+^ solid tumour xenograft models. (**a**) Flow cytometry of CXCR4 expression in human NSCLC H1155 cells. Cells were incubated with m17 antibody, followed by secondary antibody (green histogram) or with secondary antibody alone (blue line histogram). (**b**) *In vitro* cytotoxicity (dose-response) of ADC 713, free payload and non-target ADC to H1155 cells. (**c**,**d**) *In vivo* anti-tumour activity of ADC 713 (0.5 mg/kg/dose) in a disseminated xenograft tumour model derived from H1155 cells transduced to express luciferase, N = 5/group. (**c**) Kinetics of whole-body tumour burden monitored through luciferase activity imaging, SEM = standard error of the mean, ****P < 0.0001 (two-way ANOVA with Sidak’s multiple comparison test). Non-target ADC was dosed on day 9 (0.5 mg/kg) and ADC 713 was dosed twice (0.5 mg/kg/dose), on days 9 and 30 (arrows). Mice in control group were euthanized at day 26 (humane end-point) and therefore did not receive a second dose. (**d**) Kaplan-Meier analysis of median survival. (**e–g**) Micrographs of anti-CXCR4 immunohistochemistry (brown staining) in tumour cells of H1155-derived tumour xenografts (efficacy study shown in **c**,**d**). Tumours were harvested from liver at humane end-point for respective treatment cohort. (**e**,**f**) Tumour tissue of mice treated with control non-target ADC (harvested on day 26) at low (**e**) and high (**f**) magnification. (**g**) Tumour of mice treated with anti-CXCR4 ADC 713 (harvested on day 61). (**h**) Flow cytometric analysis of cell surface expression of human CXCR4, CD166 and CD44 in tumours of patient-derived xenograft TM00226 in NSG mice. Live cells were gated on human CXCR4^+^ tumour cells, and then further gated on CD166^+^CD44^+^ cells. Triple stained = samples stained for all 3 markers, FMO = fluorescence minus one gating control. Data shows mean frequency ± standard deviation, N = 4 independent tumours. **i**, *In vivo* anti-tumour activity of ADC 713 in the patient-derived xenograft TM00226 as monotherapy or in combination with gemcitabine chemotherapy, N = 5 mice/group. Black arrows = ADC dosing (0.5 mg/kg/dose), green arrows = gemcitabine dosing (75 mg/kg/dose), *P = 0.04 at day 117 (two-way ANOVA with Sidak’s multiple comparison test).

Having determined that ADC 713 has superior TI in mice, we next explored whether this ADC also displays antineoplastic activity in solid tumours. The H1155 is a non-small cell lung cancer (NSCLC) cell line derived from lymph node metastasis that expresses CXCR4^22^. We found detectable levels of CXCR4 on the cell surface of 88% of H1155 cells (Fig. 7a). ADC 713 was potent at eliminating ≈ 80% of H1155 cells *in vitro* (Fig. 7b). To investigate ADC 713’s ability to kill CXCR4^+^ H1155 cells *in vivo*, we developed a model of disseminated NSCLC, by injecting luciferase-labelled H1155 into the tail vein of NSG mice and monitoring luciferase activity signal and overt signs of disease progression. Tumours developed in lymph nodes and liver. Considering its TI in mice, ADC 713 was administered to H1155 tumour-bearing NSG mice at 0.5 mg/kg/dose, once every 21 days. The first dose caused significant, but incomplete, tumour regression and considerably extended survival (Fig. 7c,d). A second ADC 713 dose was unable to arrest tumour growth (Fig. 7c). Tumours were harvested from the liver of control ADC- and ADC 713- treated mice at respective humane end-points to test CXCR4 expression on tumour cells. Membranous CXCR4 expression was widely detected on the tumour cells from control mice (Fig. 7e,f), whereas CXCR4 expression was reduced in tumours of ADC 713-treated mice (Fig. 7g). Notably, the anti-CXCR4 antibody used for immunohistochemistry binds to an intracellular epitope and therefore it is unlikely that low detection levels of CXCR4 expression in ADC 713-treated tumours are due to epitope masking by the anti-CXCR4 ADC. Taken together with the *in vitro* CXCR4 expression in H1155 results, this data suggests that tumours developed after ADC 713 treatment arose through selection of the CXCR4^−^ subset of H1155 cells and not due to resistance of CXCR4^+^ cells to the ADC.

CXCR4 was also expressed in a subset of tumour cells from a KRAS^G13R^ mutant NSCLC patient-derived xenograft (PDX) model, featuring resistance to cisplatin and trametinib and partial resistance to paclitaxel (TM00226, The Jackson Laboratory PDX Database). We used anti-CXCR4 m17 antibody to specifically detect human CXCR4^+^ tumour cells. CXCR4 was expressed in a minority of tumour cells but, interestingly, >80% of these co-expressed the cell surface markers CD44 and CD166 (Fig. 7h), which are associated with NSCLC tumour-initiating activity^54–57^. ADC 713 dosed at 0.5 mg/kg/dose negligibly impacted tumour growth as a monotherapy. This is not surprising, given that human CXCR4 is only expressed in a minority of cancer cells and this tumour is partially resistant to the microtubule inhibitor paclitaxel. However, ADC 713 increased efficacy of gemcitabine in a treatment combination cohort (Fig. 7i). Altogether, our data indicates that lead anti-CXCR4 ADC 713 is also efficacious in NSCLC models and demonstrates high specificity towards CXCR4^+^ cancer cells.

## Discussion

ADCs hold promise for treatment of haematological malignancies, as several of these cancers harbour lineage-restricted antigen expression, thereby enticing use of potent toxins as payloads^58,59^. However, toxicity to normal tissues can limit therapeutic application. Our studies demonstrate, for the first time, that anti-CXCR4 ADCs may offer enhanced therapeutic benefit over existing anti-CXCR4 antibody therapies to aggressive haematological cancers and provide benefit to solid tumours containing CXCR4^+^ cancer cells. Previous anti-CXCR4 agents either eliminate all (cancer and normal) CXCR4^+^ cells due to high affinity coupled to inhibition of survival signalling downstream CXCR4 blockade and/or active antibody Fc-mediated effector function or cause tumour and leucocyte mobilization, due to antagonism of CXCR4-mediated retention in bone marrow^15,29,35,36,60^. The lead ADC presents a novel MoA that: 1) limits tumour and leucocyte mobilization, 2) reduces antigen-dependent ADC clearance (as shown by the enhanced exposure in HuCXCR4KI mice) and 3) mitigates toxicity to quiescent CXCR4^+^ normal tissues, including HSC/LSK progenitors. The low binding of ADC 713 contrasts with the higher estimated affinities of anti-CXCR4 therapeutic antibodies^15,35,36,60^.

Anti-CXCR4 blocking antibodies with active Fc-mediated effector function are expected to be more efficacious than ones lacking it, because of dual MoA^15,60^. However, in the case of anti-CXCR4 ADCs, we found that ADCs with active Fc-mediated effector function (ADCs 712 and 714) were less efficacious *in vivo* than ones with effector-reduced Fc (ADCs 711 and 713). Our data suggests this is due to shorter exposure of ADCs 712 and 714. Several factors are known to modulate ADC pharmacokinetics, including: isoelectric point, target-mediated drug disposition (TMDD), interaction with neonatal Fc receptor (FcRn) and site of linker-payload attachment on the antibody^45,61^. Indeed, ADCs 712 and 714 also differ from their respective counterparts with effector-reduced Fc in one different conjugation site. Another possible explanation is the existence of a second, active Fc-dependent, ADC clearance mechanism. Though the Fc:Fcγ receptor interaction should not impact ADC pharmacokinetics, abnormally short serum half-life of ADCs and antibodies has been reported in NSG mice^61,62^. TMDD is not different between ADCs of the same affinity level and the theoretical isoelectric point of all anti-CXCR4 ADCs is very similar. The ADC pharmacokinetic profiles, generated through anti-payload and anti-human IgG assays overlapped for all conjugates, therefore excluding the possibility of ADCs 712 and 714 being unstable.

With the majority of normal adult CXCR4^+^ cells being quiescent, a tubulin polymerization inhibitor as payload enhances selective killing of highly proliferative tumour cells, thereby reducing normal tissue toxicity and improving TI. Given the role of CXCL12/CXCR4 in maintaining HSC quiescence^10^, it is also possible that the higher cell binding ADC 711 is able to block CXCR4 in HSCs in spite of their low CXCR4 expression levels. The consequent increase in HSC proliferation could contribute to the observed higher HSC/LSK numbers in ADC 711-treated mice. Due to potential mitogenic effects of ADC 711 on HSC/LSK, as well as differences in cell binding between ADCs 711 and 713, we anticipate that the kinetics of recovery in bone marrow cellularity upon 3 mg/kg/dose might differ between ADC 711 and ADC 713. Another site-specific, non-cleavable linker auristatin-based anti-CXCR4 ADC has been published^40^. Though the isotype and Fc format of its backbone IgG were not reported, its binding EC_50_ on CXCR4^High^ cells is estimated ≈10-fold higher than that of h17-NV.TS, the antibody backbone for ADC 713. Even though the published ADC is a DAR2 molecule, after two doses at 2.5 mg/kg/dose it caused toxicity in HSC/LSK cells. In contrast, our low cell binding DAR4 ADC 713, dosed three times at 4.5 mg/kg/dose spared HSC/LSK populations, allowing prospective repopulation of downstream lineages affected by the ADC. This difference in toxicity profile between the two ADCs is likely due to a combination of dosing schedule, low CXCR4 expression in HSC/LSK cells and relatively lower cell binding of ADC 713. The sparing of HSC/LSK populations by ADC 713 represents a considerable advancement among CXCR4-targeting therapeutics. On the other hand, we demonstrated that DAR4 conjugates provide enhanced anti-tumour efficacy as compared to DAR2 ones in most disease models and, particularly, in AML. In principle, site-specific conjugation allows the generation of ADCs with favourable pharmacokinetics and TI at DAR ≤ 8^63^. However, due to widespread expression of CXCR4 in haematopoietic cells, on-target, but off-tumour toxicity would remain a concern. Our data indicates that ADC internalization is not affected by antibody affinity, but it is affected by cell type-intrinsic mechanisms, which still has implications on ADC design. For instance, though PBMCs showed a slower CXCR4:antibody internalization rate relatively to cancer cells, one could expect that increases in DAR can significantly augment payload delivery and thereby, toxicity to normal CXCR4^+^ tissue. Our work is the first to determine optimal anti-CXCR4 ADC design towards maximizing TI in haematological cancers.

The anti-tumour activity of anti-CXCR4 ADCs showed peculiarities likely related to CXCR4 biology in haematopoietic cells. For instance, efficacy of anti-CXCR4 ADCs did not correlate with antigen density on tumour cells: all cell lines with detectable surface CXCR4 demonstrated sensitivity to anti-CXCR4 ADCs. ADC activity in CXCR4^Low^ tumours was also observed *in vivo* (ex: OPM2 xenografts) and in these models CXCR4 expression *in vivo* remains as low as *in vitro* (Supplementary Fig. 3i). A possible explanation for this is that signalling downstream PI-3-kinase pathway (often hyper-activated in haematological malignancies^64^) inhibits endosomal sorting of CXCR4 to lysosome, instead favouring its recycling to plasma membrane, thereby decreasing ADC processing and uncoupling CXCR4 surface expression from ADC cytotoxicity^65^. In another example, our data suggests that tumour type susceptibility to anti-CXCR4 ADCs is also determined by tumour anatomical location and associated CXCL12 levels. *In vitro*, CXCL12 levels like those found in MM-diseased bone marrow enhanced ADC cytotoxicity. It is unlikely this result is due to increased CXCR4 levels in plasma membrane in the presence of CXCL12. On the contrary, CXCL12 induces CXCR4 internalization in multiple cell types^66^. Indeed, CXCL12 and bivalent antibodies have different effects on CXCR4 recycling and processing. Crosslinking by bivalent antibodies induces CXCR4 endocytosis but rapid recycling to plasma membrane. In contrast, CXCL12-mediated CXCR4 internalization enhances sorting to late endosome/lysosome^67,68^. Given that our data shows the ADC blocks CXCL12 activation of G_i_ proteins, we interpret our results as enhanced lysosome targeting of ADC:CXCR4 complexes by the presence of membrane adjacent or hetero-dimerized CXCL12:CXCR4 complexes, and thereby more efficient ADC processing. Therefore, the anti-tumour activity of anti-CXCR4 ADCs might be enhanced in the bone marrow and in other organs with high CXCL12 expression.

Though we showed that anti-CXCR4 ADCs block CXCR4 signalling, our data also suggests that payload-dependent cytotoxicity is the main mechanism mediating efficacy of anti-CXCR4 ADCs. First, ADC 513 caused increased phosphorylation of histone-H3^Ser10^ in AML blasts *in vivo* with minimal leucocytosis; second, the unconjugated antibody of ADC 711 (h17-NA) blocks CXCR4 but does not inhibit MOLP-8-derived tumour growth; third, the selective toxicity of ADCs 711 and 713 to specific CXCR4^+^ normal cells can be attributed, at least in part, to differences in cell proliferation rates (our data and^42,43^). Our data and the literature suggest that multiple factors may contribute to determine anti-CXCR4 ADC cytotoxicity to normal and cancerous CXCR4^+^ cells, beyond differences in relative CXCR4 expression levels: mitotic index, rates of CXCR4:ADC complex internalization and processing, CXCL12 levels in tumour microenvironment, expression/activity of multi-drug resistance ABC transporters and presence of genetic alterations impacting lysosome sorting pathway. Through empirical research of various ADC configurations, it was nevertheless possible to obtain an optimal anti-CXCR4 ADC presenting favourable TI. Interestingly, though AML cells’ requirement for DAR4 correlated with lower sensitivity to auristatins, we could not attribute the latter to either lower proliferation rates or higher activity of multi-drug resistance ABC transporters, as compared to other tumour types.

The lead ADC 713 is also efficacious in NSCLC solid tumour models and we expect to observe its efficacy in more solid tumour types containing CXCR4^+^ cells^23^. Given the often metastatic and tumour-initiating activity of CXCR4^+^ cancer cells, inclusion of anti-CXCR4 ADC as part of the therapy regimen might increase benefit, particularly in tumours sensitive to microtubule inhibitor agents. Targeting CXCR4 in solid tumours has been proposed as a therapeutic strategy, with the introduction of small molecule/peptide/antibody CXCR4 antagonists in clinic^9,21,69^. However, there is evidence that CXCR4 blockade *per se* might cause deleterious mobilization of both cancer cells and tumour-promoting, infiltrating stromal/immune cells^70,71^. It is therefore possible that CXCR4 blockade-mediated cell mobilization mechanisms contribute to the so far disappointing outcomes of targeting CXCR4 in solid tumours^33^. As discussed above, anti-CXCR4 ADC rather eliminates tumour cells, thereby overcoming such limitation. Our data indicates that due to ectopic and heterogeneous CXCR4 expression in solid tumour cancer cells and the specific cytotoxicity of ADC 713 to CXCR4^+^ cells, a therapeutic combination strategy is warranted. However, anti-CXCR4 ADC may also target various cell types infiltrating the tumour microenvironment recruited through the CXCL12/CXCR4 pathway and that support tumour growth, such as bone marrow-derived mesenchymal, vascular and myeloid cells^9^. Given that ADC 713 does not bind mouse CXCR4, this hypothesis has not been tested here with the use of xenograft models. Therefore, while the expression of human CXCR4 in host tumour-infiltrating cells may contribute to increase ADC 713 TMDD, it is possible that the global anti-tumour activity (i.e., towards *both* cancer cells and their supporting stroma) of ADC 713 in solid tumours was underestimated in our studies. The specific effects of anti-CXCR4 ADCs in the solid tumour microenvironment are the subject of future studies.

In conclusion, we discovered an anti-CXCR4 ADC, active in haematological and solid tumours and presenting a favourable TI. Featuring a unique MoA, ADC 713 has more limited normal tissue toxicity profile than predicted from CXCR4 expression pattern, preserving haematopoietic stem cells and progenitors. Our work in preclinical mouse models provides proof-of-concept that through empirical ADC design, it is possible to improve the TI for targets with broad normal tissue expression.

## Methods

### Antibody generation

Monoclonal mouse anti-CXCR4 antibodies were generated through hybridoma technology, using CHO cells expressing full-length human CXCR4 as immunogen. The selected antibody variable domains were cloned into human IgG1/kappa expression vectors to create the parental chimeric antibody, which was humanized by CDR grafting. Affinity maturation and sequence optimization led to generation of multiple humanized antibody variants.

### F(ab) production

Antibody variable domains were cloned into expression vectors containing 10X His tag sequences and were purified using HisTrap Excel (GE Healthcare life Sciences).

### Dissociation rate constant measurement by Surface Plasmon Resonance

Experiments were performed on a Biacore T200 Surface Plasmon Resonance biosensor (GE Lifesciences). A CaptAvidin (ThermoFisher Scientific) sensor chip was prepared at 25 °C with a running buffer of 10 mM HEPES, 150 mM NaCl, 0.05% Tween-20, pH 7.4. All surfaces of a Biacore CM3 sensor chip were activated with a 1:1 (v/v) mixture of 400 mM EDC and 100 mM NHS for 7 min., at a flow rate of 10 µL/min. CaptAvidin was diluted to 20 µg/mL in 10 mM sodium phosphate pH 7.5 and injected on all flow cells for 7 min. at 20 µL/min. All flow cells were blocked with 1 M ethanolamine pH 8.5 for 7 min. at 10 µL/min. All interaction analysis was performed at 37 °C using a running buffer of 10 mM HEPES, 150 mM NaCl, 0.05% Tween-20, pH 7.4, 1 mg/mL bovine serum albumin (BSA). All reagents were diluted into running buffer for the analysis. In each analysis cycle, 10 µg/mL biotinylated wheat germ agglutinin was captured on all flow cells for 5 min. at 5 µL/min. Then, 0.43 Units/mL of CXCR4 lipo-particles (Integral Molecular) were captured onto flow cell 2, and 0.43 Units/mL of NULL lipo-particles (Integral Molecular) were captured onto flow cell 3. After capture of lipo-particles, analyte (buffer, or 3.3 nM anti-CXCR4 F(ab)) was injected for 3 min. at 30 µL/min and dissociation was monitored for 10 min. At the end of the analysis cycle, the surfaces were regenerated with four 60-second injections of 0.1 M glycine pH 12, 0.1% Triton X-100 at 10 µL/min. Double-referenced sensorgrams were fit to a 1:1 Langmuir with mass transport model using Biacore T200 Evaluation Software 2.0.

### ADC production

Antibodies were transiently expressed in Expi293 cells and purified using Protein-A chromatography. Antibodies were site-specifically conjugated with AcLys-VC-PABC-Aur0101 or AmPEG6C2-Aur0131^47,72^, to engineered glutamine-containing sites using microbial transglutaminase, as previously described^44,45^. These sites were: site C, insertion of LLQG tag at position 135 in the heavy chain; site D, replacement of residue 447 with LLQGA tag in the heavy chain; site F, insertion of GGLLQGPP tag after residue 214 in the light chain; site G, N297A deglycosylation mutant where conjugation happens at native Q295 on the heavy chain; site H, N297Q mutant where conjugation happens on Q295 and Q297 on the heavy chain. The AcLys-VC-PABC linker was selected based on our previous work as being one of the most stable linkers towards cleavage by carboxylesterase 1C at the selected positions^46^. Briefly, antibody concentration was adjusted to 5 mg/mL in buffer containing 25 mM Tris-HCl, pH 8.5 (for AmPEG6C2-Aur0131) or pH 8.0 (for AcLys-VC-PABC-Aur0101), 100 mM sodium chloride. Linker-payload was added in a 10-fold or 20-fold molar excess over antibody for DAR2 and DAR4 conjugation, respectively. The conjugation reaction was initiated by addition of 2% (weight/vol) bacterial transglutaminase (Ajinomoto Activa TI) and incubated with gentle shaking at 37 °C for 16 hours. The reaction mixture was adjusted to 0.75 M NH_4_SO_4_, 25 mM KH_2_PO_4_, pH 7 (buffer A), and the material was applied to a HiTrap Butyl Sepharose High Performance column (GE Healthcare), washed with 5 column volumes of buffer A, and eluted with a linear gradient over 20 column volumes into 25 mM potassium phosphate, pH 7. Fractions containing the conjugate were pooled, dialyzed into PBS, concentrated using a 10-kDa Amicon Ultra centrifugal filter unit (Millipore), and sterilized with 0.2 μm filter. DAR for all ADCs was determined by LC/MS and diphenyl analysis. Non-target isotype ADCs (NNC) with same conjugation as anti-CXCR4 ADCs served as a specificity control.

### Bioassay of CXCR4 signalling blockade

Antibodies and ADCs were probed for antagonist activity using Hit Hunter cAMP assay (DiscoverX).

Briefly, CHO-K1 cells over-expressing human CXCR4 were seeded in a total volume of 20 μL into white walled, 384-well microplates and allowed to attach at 37 °C. Five microliters of test antibodies in a 3-fold dilution series in cAMP Assay Buffer (final concentration range: 0.004–267 nM) were added to cells and incubated at 37 °C for 30 min. Five microliters of a mixture of 6X EC_80_ CXCL12 + EC_80_ forskolin (determined in pilot experiments) was added to cells and incubated at 37 °C for 30 min. Assay signal was generated through incubation at room temperature after addition of 20 μL cAMP XS + ED/CL lysis cocktail for 1 hour, followed by incubation with 20 μL cAMP XS + EA reagent for 3 hours. Microplates were read in a PerkinElmer EnvisionTM instrument for chemiluminescent signal detection. Data was analysed using CBIS data analysis suite (ChemInnovation).

### Cell culture and reagents

Cell lines were purchased from ATCC or DSMZ and cultured in RPMI medium1640 (Corning) with 10% foetal bovine serum (FBS, Corning). Cell lines were determined to be mycoplasma and pathogen free prior to use (IDEXX BioResearch). PBMCs were isolated from buffy coats (obtained from Stanford University’s IRB approved blood donor program) through a Ficoll-Paque density gradient, followed by RBC lysis with ACK lysing buffer and two washes in PBS. Unprocessed human bone marrow (obtained from Lonza’s IRB approved bone marrow donor program) was washed in PBS, then erythrocytes lysed with ACK lysing buffer (Gibco). Prior to all experiments involving cells, live cell density and viability were determined in a Vi-Cell instrument (Beckman Coulter). Bortezomib was purchased from SelleckChem. Gemcitabine (GEMZAR) was purchased from Eli Lilly and Company.

### *In vitro* cytotoxicity and cell proliferation assays

Tumour cells were seeded in white-walled, clear-bottom 96-well plates (Corning), at 10,000/well in 100 µL RPMI medium 1640 + 10% FBS. The next day, 25 µL of RPMI medium 1640 with 4-fold serial dilutions of ADCs (final concentration range: 0.004–267 nM) or free payload (final concentration range: 0.0006–40 nM) were added in triplicate. After 4 days incubation, viable cells were quantified using CellTiter-Glo Luminescent Viability Assay (Promega) in a SpectraMax M5 plate reader (Molecular Devices). The data was analysed using SoftMaxPro and curve fitted in GraphPad Prism 7 using non-linear regression (*log(inhibitor) vs*. *normalized response-variable slope*) to determine IC_50_. In some experiments, 100 ng/mL recombinant human CXCL12 (R&D systems) was spiked into ADC dilution media prior to cell incubation, in order to mimic ADC vs. ligand competition for CXCR4 in bone marrow. In other experiments, recombinant human CXCL12 was added to cells at different concentrations (100, 7 and 2.4 ng/mL) immediately before addition of ADC (10, 5 and 2 nM). For payload screening, free drugs were solubilized in DMSO, then serial diluted (final concentration range: 0.0006–40 nM) in RPMI medium 1640 as described above for ADC cytotoxicity assay. Ten thousand tumour cells or 50,000 normal PBMCs were seeded in black-walled, clear bottom 96-well plates (Corning) in 100 µL RPMI medium 1640 + 10% FBS or X-Vivo media (Lonza), respectively, and incubated with free payloads for 48 hours. Cell death was quantified at end-point using CellTox Green Cytotoxicity Assay (Promega) in a SpectraMax M5 plate reader (Molecular Devices). To compare tumour cell line-intrinsic differences in cell proliferation, 10,000 cells in 100 µl RPMI medium 1640 + 10% FBS were seeded in flat-bottom 96-well plates. XTT (Cell proliferation kit II, Roche) was added immediately and incubated for 4 hours (time 0) or for the last 4 hours of time points 24, 48 and 72 hours post cell seeding. Absorbance was measured in a SpectraMax 250 (Molecular Devices).

### *In vivo* efficacy and tolerability

All animal procedures were conducted in an AAALAC-accredited facility, in accordance with the US National Research Council Guide for the Care and Use of Laboratory Animals. Animal use protocols were reviewed and approved by the Pfizer Institutional Animal Care and Use Committee. For efficacy studies, two types of xenografts were used in female mice aged 6–7 weeks: subcutaneous and orthotopic/disseminated. For subcutaneous xenografts, tumours were implanted into the lateral right flank, measured on the two largest dimensions using callipers and tumour volume calculated (*volume* = *length* × *width*^2^ *×* *0*.*5*). C.B.-17 SCID beige mice (Taconic) were inoculated with 5 × 10^6^ Ramos cells suspended in 100 µL PBS. When tumour volume reached ≈ 265 mm^3^, mice were randomized and received 3 mg/kg (single dose) ADC through the tail vein. Tumour fragments (1–2 mm^3^) of the NSCLC PDX model TM00226 (The Jackson Laboratory) were implanted in NSG mice (The Jackson Laboratory). When tumour volume reached ≈ 250 mm^3^, mice were randomized and received ADC (0.5 mg/kg) and/or gemcitabine (75 mg/kg) through the tail vein at the time points indicated in figure. Gemcitabine was chosen for combination with ADC because it causes partial tumour growth inhibition in this PDX model^73^. For orthotopic/disseminated xenografts, cells stably expressing luciferase-2A-GFP (Amsbio) and suspended in 100 µL PBS were inoculated through the tail vein of NSG mice (The Jackson Laboratory). The implanted cell number was model dependent and titrated in pilot experiments, so that untreated animals reached humane end-point within 3–8 weeks. ADCs were administered through the tail vein at the doses indicated in figure. Whole body tumour burden (expressed as *photon count* × *sec*^*−*1^) was measured in an IVIS SpectrumCT (PerkinElmer), 10 min. after intra-peritoneal injection of 3 mg (200 µL) luciferin sodium salt (Regis Tech.) and using the minimum target of 30,000 luminescent counts and automatic exposure as data acquisition parameters. Individual time to humane end-point was plotted into GraphPad Prism 7 to calculate median survival by Kaplan-Meier analysis with log-rank (Mantel-Cox) test. To investigate toxicity/tolerability, a repeat dose study was conducted in male and female HuCXCR4KI mice of 12–13 weeks age^8^. We defined tolerability as the maximum tested dose that would not cause more than 20% body weight loss and/or moribundity/mortality in >90% of mice upon 3 doses, once every 3 weeks. Necropsies were performed 3 days after last dose (or at humane end-point) and tissues were processed for haematology, histology and flow cytometry.

### Flow cytometry

The same live cell number or whole blood volume was stained across samples within each experiment. Except where stated otherwise, each antibody was used at 1 µg per 1 × 10^6^ live cells. For *ex vivo* analysis, single cells were first washed in PBS and incubated with LIVE/DEAD Fixable Blue Dead Cell Stain kit (Life Tech.) Incubations with viability dye and antibodies on non-fixed cells were performed at 4 °C (20 min.) and all buffers were kept ice-cold. Primary antibodies to intracellular antigens were incubated at room temperature (45 min.). In cell binding assays, 2 × 10^5^ cells were incubated with serial diluted F(ab) or IgG, then with anti-human IgG, F(ab’)_2_ fragment-specific AlexaFluor647-conjugated goat F(ab’)_2_ (Jackson ImmunoResearch). An MFI-concentration curve was plotted in GraphPad Prism 7 for EC_50_ determination: *log(agonist) vs*. *response – variable slope (four parameters)*. Cell surface CXCR4 density in human cell lines and normal human haematopoietic cells was estimated with 12G5-PE antibody (BD Biosciences) binding (20 µL antibody per 2 × 10^5^ cells for tumour cell lines and 20 µL antibody per 1 × 10^6^ nucleated cells for normal human bone marrow) and Quantibrite-PE calibration beads (BD Biosciences). Human P-glycoprotein 1 detection in 2 × 10^5^ tumour cell lines and normal PBMCs was with UIC2-APC antibody (eBioscience), following manufacturer’s instructions. Nucleated human bone marrow cells were washed in PBS-0.5% BSA and a total of 5 × 10^6^ cells were stained with anti-CXCR4-PE (clone 12G5), anti-CD34-APC (clone 8G12, BD Biosciences) and anti-CD45-FITC (clone 2D1, R&D Systems). Femur bone marrow of MV4–11-tumour-bearing mice was flushed in PBS. MV4–11 tumour cell apoptosis was analysed *ex vivo* using anti-human CD45-APC-Cy7 (clone 2D1, BD Biosciences) and anti-human CD33-BV421 (clone WM53, BD Biosciences) antibodies and the Apoptosis detection kit APC (eBiosciences) on 1 × 10^6^ cells. For *ex vivo* quantification of histone H3^Ser10^ phosphorylation in MV4–11-derived xenografts, bone marrow cells (1 × 10^6^) were stained with anti-human CD45-APC-Cy7 (clone 2D1, BD Biosciences) and anti-human CD33-BV421 (clone WM53, BD Biosciences) antibodies, then fixed and processed for nuclear protein staining with anti-histone H3^Ser10^-phospho-specific-AlexaFluor488 antibody (clone 11D8, Biolegend). Leucocyte mobilization assay was performed as described^8^. For analysis of erythroid and leucocyte progenitors respectively, a total of 1 × 10^6^ and 5 × 10^6^ cells were stained with live/dead fixable blue (Life Tech.), then for cell surface markers^74–76^. For *in vivo* analysis of cell proliferation in homeostatic conditions, HuCXCR4KI mice received 0.5 mg EdU (Life Tech.) in PBS (5 mg/mL) through intra-peritoneal injection, 60 and 30 min. before sacrifice. Femur bone marrow was flushed and cells from EdU-pulsed mice were stained for cell surface markers, as above, and then processed with Click-it Plus EdU-AlexaFluor647 Cell Proliferation kit (Life Tech.). Stained cells from an animal not administered EdU served as “fluorescence minus one” (FMO) control. Cell surface CXCR4 expression in haematopoietic precursors of HuCXCR4KI mice was detected with m17-AlexaFluor647 antibody conjugated using AlexaFluor647 Antibody Labelling kit (Life Tech.). Numbers of femur bone marrow cell subsets were enumerated based on total flushed live cell number (*volume* × *density*) and number of live, single cells and of population subsets recorded by the cytometer. H1155 cells were incubated with anti-CXCR4 m17 antibody, followed by AlexaFluor488-conjugated anti-human Fc-specific antibody or with AlexaFluor488-conjugated anti-human Fc-specific antibody (Jackson ImmunoResearch) alone as control. The PDX tumour TM00226 was cut in small fragments and processed into single cells using the Tumor Dissociation Kit, mouse and GentleMACS Octo Dissociator (Miltenyi Biotec). Cells were washed in PBS-0.5% BSA and a total of 5 × 10^6^ cells were stained with anti-CXCR4 m17-AlexaFluor647 antibody, anti-human CD44-BV786 (clone G44–26, BD Biosciences) and anti-human CD166-AlexaFluor488 (clone 105902, Invitrogen). Gating and corrected geo MFI (background MFI of control sample subtracted from MFI of antibody –stained sample) were defined based on FMO controls in all experiments, except for measurement of CXCR4 surface density with 12G5-PE antibody in tumour cell lines and of P-glycoprotein 1 with UIC2-APC antibody in tumour cell lines and normal PBMCs, in which the respective unstained cells were used as auto-fluorescence controls. The percentage of CXCR4^+^ H1155 cells was determined based on signal of cells stained with AlexaFluor488-conjugated anti-human Fc-specific antibody alone. The activity of MDR1 (P-glycoprotein 1), MRP1/2 and BCRP in tumour cell lines *in vitro* was measured using the MDR assay kit (Abcam). Samples were run in LSRII or Fortessa cytometers (BD Biosciences) and data analysed using FlowJo v10.

### Haematology

Peripheral blood cell counts were determined in an Advia-2120 analyser (Siemens Healthcare Diagnostics).

### Pharmacokinetics

Mice were dosed with ADCs through the tail vein. Blood samples were collected using a serial sampling protocol. Blood was drawn via tail vein puncture and 10 µL of whole blood mixed with 190 µL of cold buffer (0.2 M Tricine, 0.15 M NaCl, 3 mM EDTA, 1% BSA, 0.1% Tween-20, 0.05% Proclin300, pH 8.5). Samples were centrifuged at 1500 × *g* for 10 min. and the supernatant harvested for analysis. The concentration of total human antibody (Ab) and ADC was measured by ligand binding assay (Gyrolab). A biotinylated goat anti-human IgG (H + L) (Southern Biotech) was used for capture (50 µg/mL). The detection antibodies used were: AlexaFluor647-goat anti-human IgG (H + L) (Bethyl) at 5 µg/mL and AlexaFluor647-anti-payload antibody (proprietary to Pfizer) at 2 µg/mL, for the total antibody and conjugated antibody assays, respectively. Standards and controls were spiked in mouse serum prior to dilution in Superblock buffer (ScyTek). Samples were diluted in Superblock and run in duplicate on a Bioaffy-200 CD in a three-step, two-wash method using wash buffers 1 (PBS-0.05% Tween-20) and 2 (20% ethanol-0.5% SDS). Data were regressed in Gyrolab Evaluator using a 5-parameter logistic fit. Pharmacokinetic parameters were calculated using Phoenix WinNonlin7.0.

### ELISA

Detection of CXCL12 in mouse serum by ELISA (Quantikine, R&D systems) was performed as previously described^8^.

### *In vitro* antibody-dependent cell-mediated cytotoxicity (ADCC) and complement-dependent cytotoxicity (CDC) assays

For CDC, 10,000 Daudi cells in 100 µl RPMI medium 1640 + 5% FBS were seeded in U-bottom 96-well plates. Antibodies or ADCs (7 µg/mL) and 2.5% human serum (Innovative Research) were added and cells incubated for 4 hours. For ADCC, NK cells (HemaCare) were thawed 24 hours prior to assay and maintained in X-Vivo medium (Lonza). Ten thousand MOLT-4 cells in 100 µl RPMI medium 1640 + 5% FBS were seeded in U-bottom 96-well plates and incubated with 5 µg/mL antibodies or ADCs and NK cells, for 4 hours. In both assays, each experimental condition was run in duplicate wells. Tumour cell lysis was quantified at end-point with CytoTox 96 Non-Radioactive Cytotoxicity Assay (Promega) in a SpectraMax 250 (Molecular Devices). The specific cell lysis was calculated by subtracting the background lysis levels of tumour cells + complement (for CDC assay) or tumour cells + NK cells (for ADCC assay) and then normalizing by signal from untreated cells lysed with detergent at assay end-point.

### Antibody internalization assay

For each cell type, 10,000 cells were suspended in RPMI medium1640 + 10% FBS, plated in 0.01% poly-L-ornithine (Sigma) -coated 96-well plates and allowed to attach for 1 hour. Antibodies were labelled with IncuCyte Human FabFluor-pH Red Antibody Labelling Reagent (Essen BioScience) at a molar ratio of 1:3 (test antibody: labelling Fab), following the manufacturer’s instructions. Labelled antibodies were added to cells at a final concentration of 3 µg/mL and the plate immediately transferred to an IncuCyte S3 Live-Cell Analysis System (Essen BioScience) placed inside a tissue culture incubator (37 °C and 5% CO_2_). Automated phase and red fluorescence micrographs were captured with a 20x objective within the first 5 minutes post addition of labelled antibodies to cells and then every 15 minutes for 12 hours. Each assay condition was run in triplicate wells, with 9 micrographs captured and analysed per well, per time point. When the Fab-conjugated fluorescent probe is exposed to the low pH of the lysosome, the red fluorescence area and signal intensity inside the cell increases. The total red object area (µm^2^/well) was quantified for each time point using the IncuCyte software (Essen BioScience).

### Immunohistochemistry

CXCR4 immunohistochemistry was performed using the rabbit monoclonal UMB2 antibody (Abcam), as previously described^8^, using rodent HIER pH 6.0 buffer for antigen retrieval (Biocare Medical).

## Supplementary information

Supplementary figures and tables

## Data Availability

All data and associated experimental methods are displayed in the manuscript.
