# Supplementary material for: Optimal design, anti-tumour efficacy and tolerability of anti-CXCR4 antibody drug conjugates
Source: Sci Rep. 2019 Feb 21;9:2443. doi: 10.1038/s41598-019-38745-x (PMC6384886; doi:10.1038/s41598-019-38745-x)
Supplement: Supplementary file 1 — Supplementary figures and tables [file 41598_2019_38745_MOESM1_ESM.pdf]

**Supplementary information: supplementary figures and tables**

# Optimal design, anti-tumour efficacy and tolerability of anti-CXCR4 antibody drug conjugates

Maria José Costa, Jyothirmayee Kudaravalli, Jing-Tyan Ma, Wei-Hsien Ho, Kathy Delaria, Charles Holz, Angela Stauffer, Allison Given Chunyk, Qing Zong, Eileen Blasi, Bernard Buetow, Thomas-Toan Tran, Kevin Lindquist, Magdalena Dorywalska, Arvind Rajpal, David L. Shelton, Pavel Strop and Shu-Hui Liu

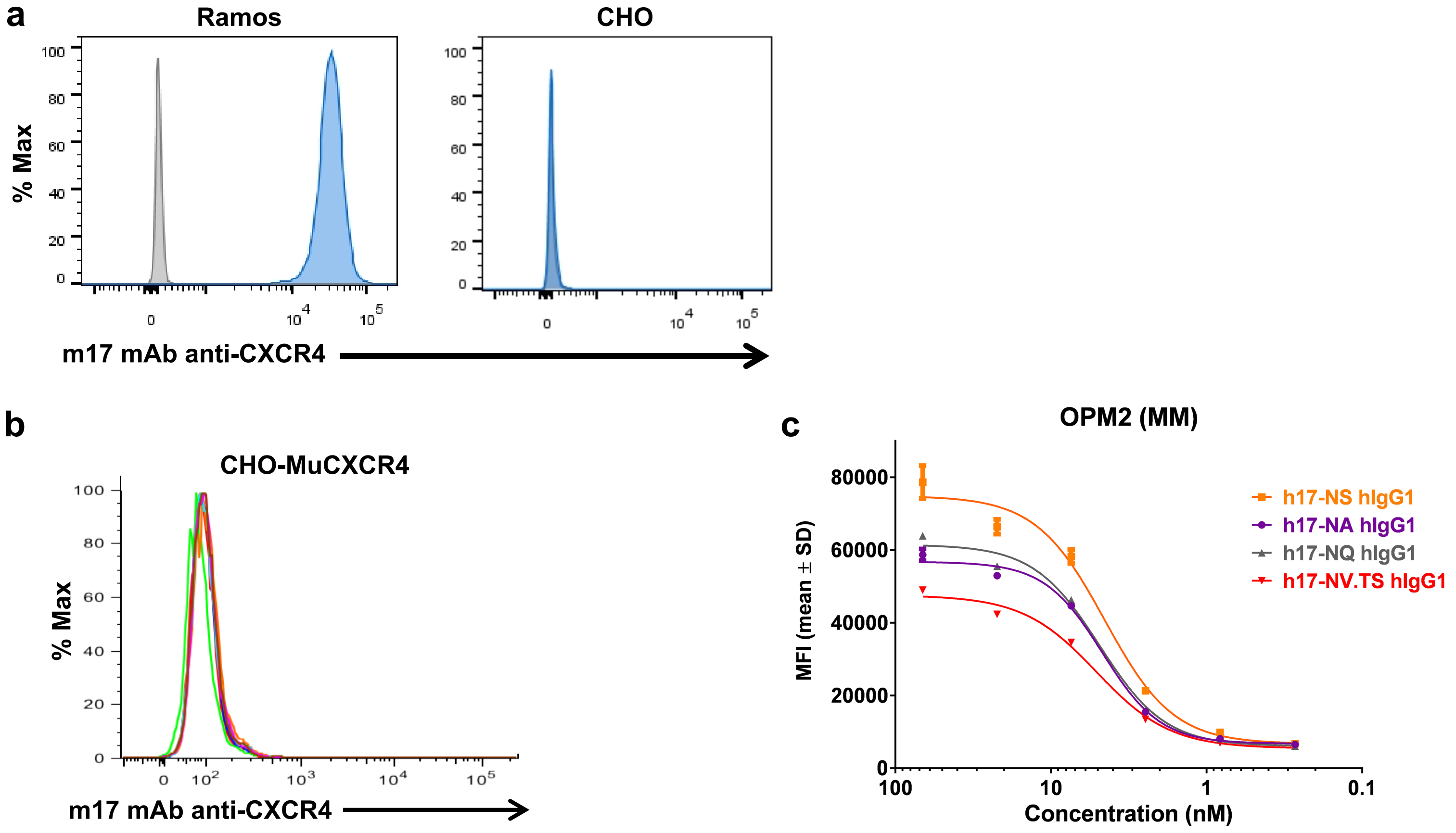


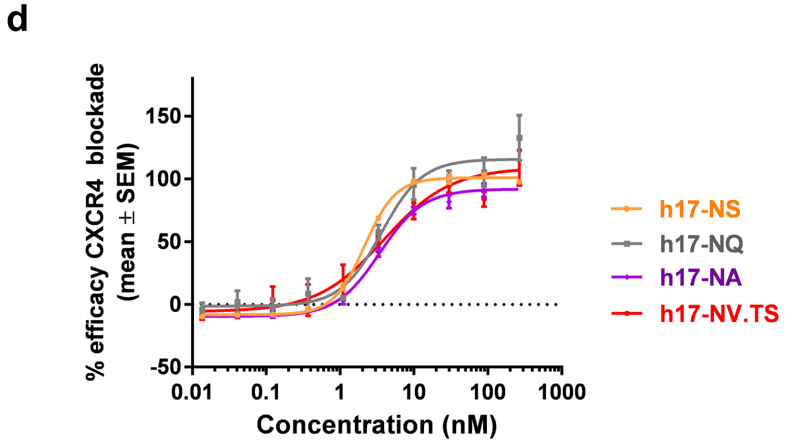


| Antibody (hIgG1) | $IC_{50}$ (nM) |
|---|---|
| h17-NS | 2.1 |
| h17-NQ | 3.6 |
| h17-NA | 3.4 |
| h17-NV.TS | 3.9 |

**Supplementary Fig. 1 (related to Fig. 1). a,** Flow cytometric analysis of m17 hIgG1 binding (blue histogram) on the $CXCR4^{High}$ Ramos cell line and on control CHO cells. Grey histogram is cells stained with secondary antibody alone. **b,** Flow cytometric analysis of m17 hIgG1 and its humanized variants binding on CHO expressing mouse CXCR4 (MuCXCR4). All histograms overlap with that of secondary antibody alone. **c,** Flow cytometric analysis: binding of serial diluted IgG on $CXCR4^{Low}$ MM-derived OPM2 cells, SD = standard deviation. **d,** Bioassay of CXCR4 blockade by h17 sequence variants on CHO-K1 cells over-expressing CXCR4, incubated in the presence of $EC_{80}$ forskolin and $EC_{80}$ CXCL12. SEM = standard error of the mean.

**Supplementary Table 1:**
**Dissociation rate constants for antibody F(ab) at 37 ºC determined by SPR**

| mAb | $k_d$ (1/s) | $t_{1/2}$ (min) | Binding on $CXCR4^+$ cells |
|---|---|---|---|
| h17-NV.TS | 5.10E-03 | 2.3 | Low |
| h17-NA | 4.20E-03 | 2.8 | Medium |
| h17-NQ | 2.20E-03 | 5.2 | Medium |
| h17-NS | 4.30E-04 | 26.8 | High |
| m17 | 1.30E-03 | 8.7 | High |

$k_d$ = dissociation rate constant. $t_{1/2}$ = dissociation half life (calculated from $k_d$).

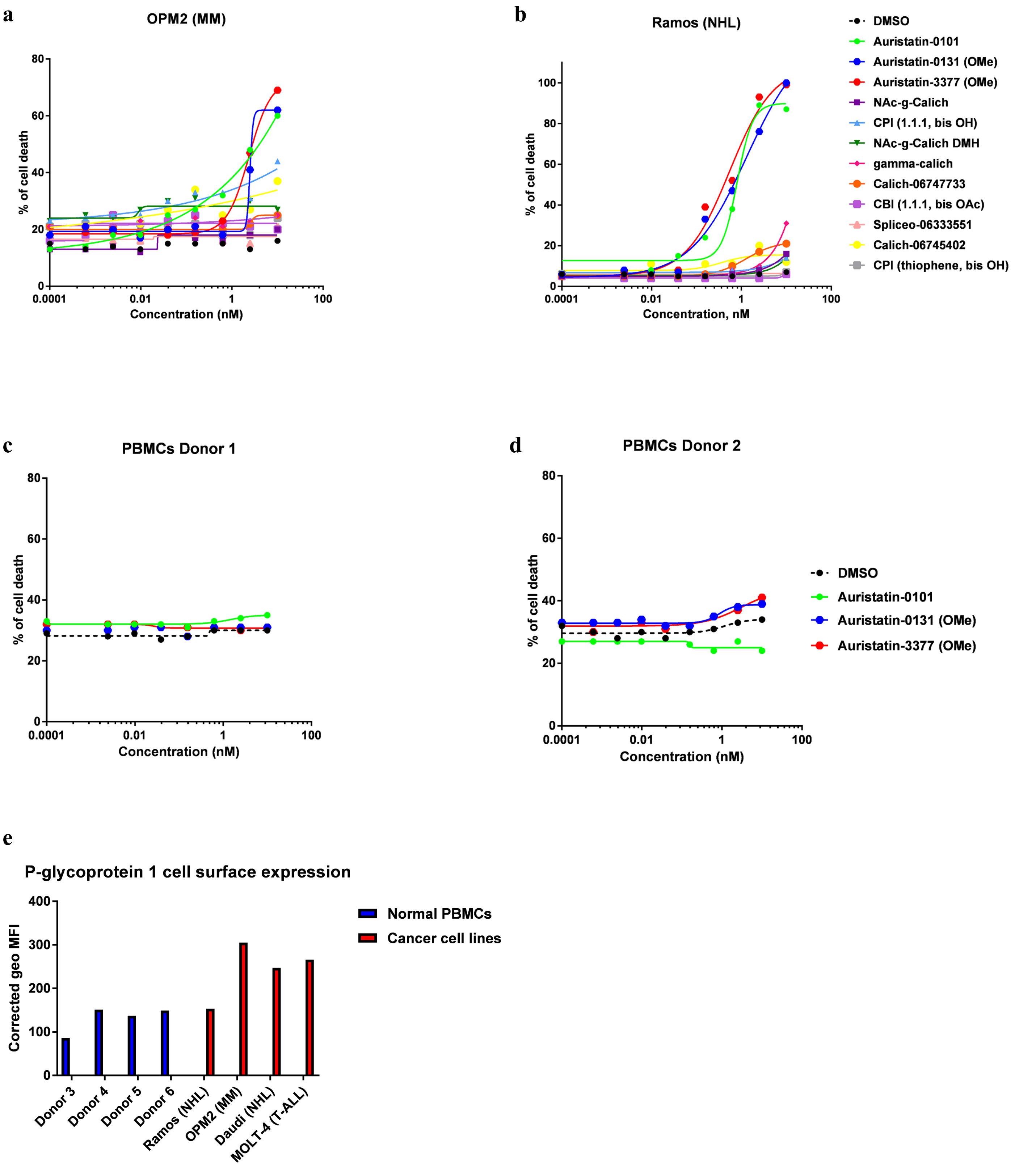


**Supplementary Fig. 2. Auristatins cause cytotoxicity on cancer cell lines, but not on normal PBMCs.** Cells were incubated with serial diluted payloads as free drugs (or as a modified cell membrane permeable form -OMe) or with vehicle control for 48 hours. Cumulative cell death was measured at endpoint using CellToxGreen Assay (Promega). Data was plotted in GraphPad Prism 7 as non-linear fit of log(concentration) vs. response. **a-b,** Screening of free payloads on tumor cell lines. **c-d,** Cytotoxicity of auristatins on normal PMBCs. **e**, Expression of P-glycoprotein 1/ABCB1 in normal PBMCs and cancer cell lines. Cells were incubated with APC-conjugated anti-P-glycoprotein 1 antibody (clone UIC2) and analyzed by flow cytometry. Geo MFI of the respective cell type unstained control was subtracted from the geo MFI of antibody stained samples (corrected geo MFI) to allow for comparison of P-glycoprotein expression across cell types with different levels of auto-fluorescence.

**Supplementary Table 2. Relative CXCR4 density on plasma membrane of haematological cancer-derived cell lines and normal hematopoietic cells.** Number of 12G5 mAb bound/cell estimated based on MFI of total population in up to 4 independent experiments per cell type.

| Cell type | Cancer type | 12G5 mAb/Cell | | | | Average 12G5 mAb/Cell | Relative surface CXCR4 density level |
|---|---|---|---|---|---|---|---|
| Ramos | **NHL** | 30598 | 201774 | | | 116186 | **High CXCR4 (++++)** |
| HPB-ALL | **T-ALL** | 30839 | | | | 30839 | |
| NALM6 | **B-ALL** | 28576 | | | | 28576 | |
| BV173-GFP-luc | **B-ALL** | 28287 | | | | 28287 | |
| Daudi | **NHL** | 28480 | 3270 | | | 15875 | |
| Jurkat | **T-ALL** | 7523 | 18414 | 13788 | | 13242 | |
| JEKO-1-GFP-luc | **NHL** | 11914 | | | | 11914 | |
| Molp8-GFP-luc | **MM** | 9214 | 9041 | | | 9128 | **Medium-high CXCR4 (+++)** |
| REH | **ALL** | 4201 | | | | 4201 | |
| Raji-GFP-luc | **NHL** | 3841 | | | | 3841 | |
| H929-VR20-GFP-luc | **MM** | 4038 | 3184 | | | 3611 | |
| U937 | **AML** | 3439 | 3142 | | | 3291 | |
| U266-GFP-luc | **MM** | 2417 | 2062 | | | 2240 | |
| 697 | **B-ALL** | 1944 | | | | 1944 | **Medium-low CXCR4 (++)** |
| KMS12BM-GFP-luc | **MM** | 1108 | 1681 | | | 1394 | |
| OPM2-GFP-luc | **MM** | 966 | 2154 | 693 | 1011 | 1206 | |
| HL60 | **AML** | 516 | 1771 | | | 1144 | |
| MOLM13 | **AML** | 984 | | | | 984 | **Low CXCR4 (+)** |
| MM1S-GFP-luc | **MM** | 675 | 1165 | | | 920 | |
| MV-4-11-luc | **AML** | 499 | 1260 | | | 880 | |
| Normal BM $CD45^{low}CD34^{+}$ | **N/A** | 519 | 534 | | | 527 | |
| Normal BM $CD45^{+}CD34^{-}$ | **N/A** | 324 | 450 | | | 387 | |
| EOL1 | **AML** | 358 | | | | 358 | |
| ML2-GFP-luc | **AML** | 340 | | | | 340 | |
| Kasumi3 | **AML** | 146 | | | | 146 | **CXCR4 level too low, no *in vitro* cytotoxicity of anti-CXCR4 ADCs observed** |
| Kasumi1 | **AML** | 94 | | | | 94 | |
| K562 | **CML** | 59 | 67 | | | 63 | |
| H929 | **MM** | 47 | | | | 47 | |
| TF1 | **AML** | 14 | | | | 14 | |

Normal human bone marrow (BM) data is from two different donors. GFP-luc = cells transduced and selected to express high levels of GFP-luciferase for *in vivo* tracing of tumor burden.

**Supplementary Table 3.** ***In vitro*** **cytotoxicity screening of m17-derived ADCs on cell lines derived from haematological cancers.**
See also Table 1 for ADC configuration and properties and Supplementary Table 2 for estimated relative density of CXCR4 on cell surface. Data for the most efficacious ADC for each cell line is highlighted in bold.

| | $IC_{50}$ (nM) | | | | | | | | | | | | | | | | | | | | |
|---|---|---|---|---|---|---|---|---|---|---|---|---|---|---|---|---|---|---|---|---|---|
| | Ramos (NHL) | Raji (NHL) | Jeko1 (NHL) | REH (NHL) | H929-VR20 (MM) | Molp8 (MM) | OPM2 (MM) | KMS12BM (MM) | U266 (MM) | BV173 (B-ALL) | Nalm6 (B-ALL) | 697 (B-ALL) | Jurkat (T-ALL) | HPB-ALL (T-ALL) | U937 (AML) | EOL-1 (AML) | HL-60 (AML) | Molm13 (AML) | MV4-11 (AML) | K562* (AML) | Kasumi-1* (AML) |
| **NNC-358** | 180.900 | >267 | 139.500 | >267 | >267 | 165.533 | 121.500 | 127.889 | 70.793 | 57.91 | >267 | >267 | >267 | >267 | 151.700 | >267 | >267 | >267 | 220.600 | >267 | 161.700 |
| **Aur0101** | 0.208 | 0.411 | 0.031 | 0.042 | 2.035 | 0.920 | 0.210 | 0.042 | 0.029 | 0.0004 | 0.079 | 0.104 | 0.053 | 0.076 | 1.016 | 4.440 | 3.367 | 1.681 | 5.297 | 3.366 | 3.247 |
| **381** | 0.268 | 77.800 | 0.269 | 0.225 | 6.256 | 1.119 | 0.480 | 2.246 | 0.042 | 0.068 | 0.230 | 88.267 | 0.135 | 0.849 | 0.397 | 3.153 | 2.446 | 1.860 | 3.483 | 101.9 | 52.520 |
| **519** | 0.312 | 248.467 | 0.275 | 0.797 | >267 | 0.900 | 0.314 | 7.812 | 0.039 | 0.059 | 0.222 | 140.333 | 0.187 | 1.383 | 0.456 | 1.097 | 3.828 | 2.003 | 2.432 | >267 | 164.400 |
| **518** | 0.291 | 18.233 | 0.172 | 0.122 | 17.587 | 0.268 | 0.184 | **0.340** | 0.041 | 0.057 | 0.172 | 26.420 | 0.098 | 0.332 | **0.276** | **0.428** | **1.126** | 0.643 | **0.598** | 255.5 | 77.790 |
| **Aur0131 (OMe)** | 0.094 | 0.755 | 0.035 | 0.011 | 0.847 | 0.750 | 0.132 | 0.037 | 0.024 | 0.0004 | 0.094 | 0.064 | 0.095 | 0.070 | 1.377 | 4.438 | 3.232 | 1.809 | 7.396 | 4.334 | 3.448 |
| **510** | 0.217 | 142.600 | 0.167 | 0.052 | 0.662 | 0.201 | 0.126 | 176.198 | 0.018 | 0.038 | 0.232 | 214.400 | **0.037** | 0.237 | 4.453 | 0.785 | 6.726 | 0.461 | 1.129 | >267 | >267 |
| **513** | **0.128** | **7.760** | **0.104** | **0.005** | **0.228** | **0.108** | **0.038** | 0.347 | **0.008** | **0.028** | **0.113** | **0.814** | 0.043 | **0.105** | 0.997 | 0.856 | 1.592 | **0.262** | 1.040 | >267 | >267 |

*Cell lines with undetectable cell surface CXCR4 expression.

**a**

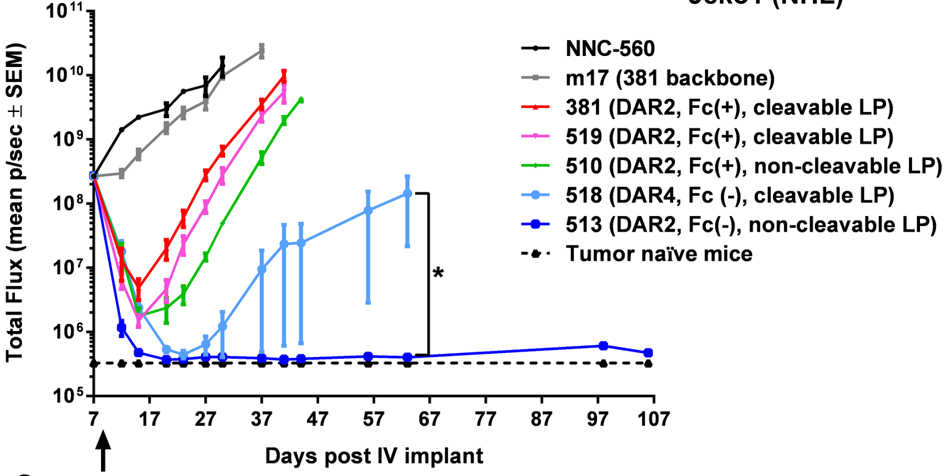


**b**

| Treatment | Survival (days) |
|---|---|
| NNC-560 | 30 |
| m17 | 37 |
| 381 | 44 |
| 510 | 44 |
| 519 | 48 |
| 518 | >98 |
| 513 | >98 |

** (510 vs 513)

**c**

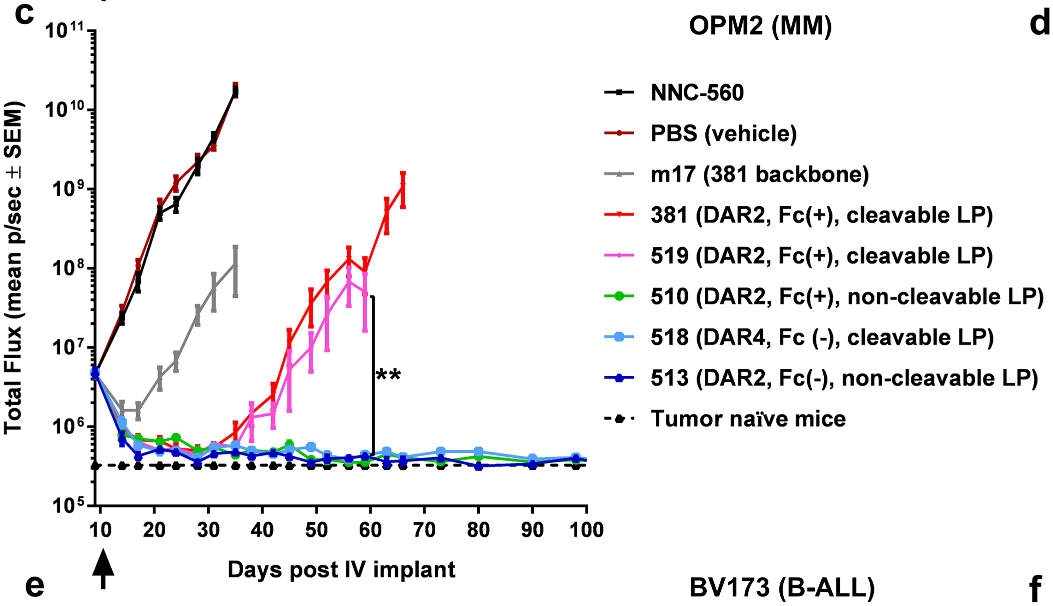


**d**

| Treatment | Survival (days) |
|---|---|
| PBS | 34 |
| NNC-560 | 34 |
| 381 | 78 |
| 519 | 90 |
| 510 | >210 |
| 518 | >210 |
| 513 | >210 |

* (519 vs 510)

**e**

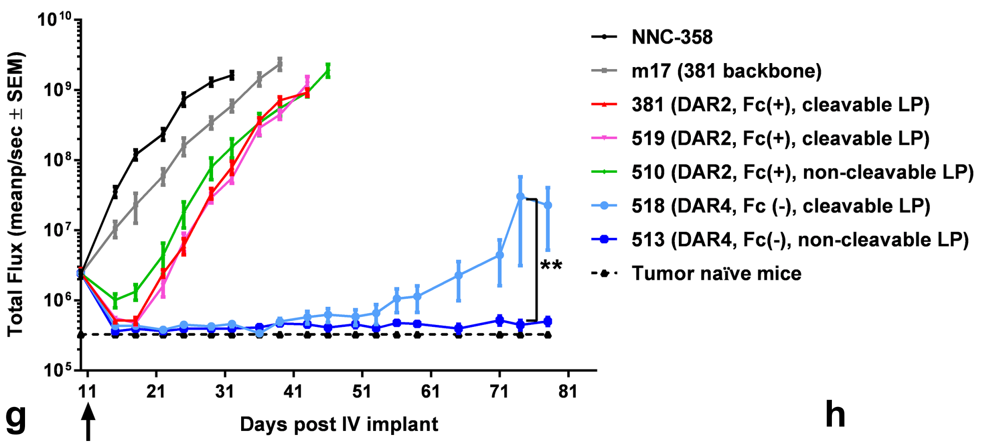


**f**

| Treatment | Survival (days) |
|---|---|
| NNC-358 | 36 |
| m17 | 43 |
| 381 | 50 |
| 519 | 50 |
| 510 | 50 |
| 518 | 120 |
| 513 | >135 |

** (518 vs 513)

**g**

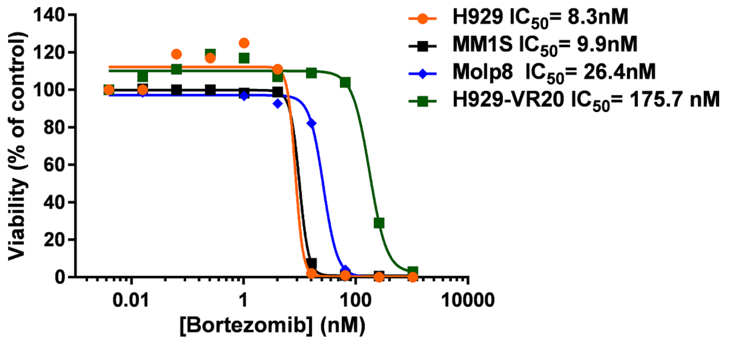


**h**

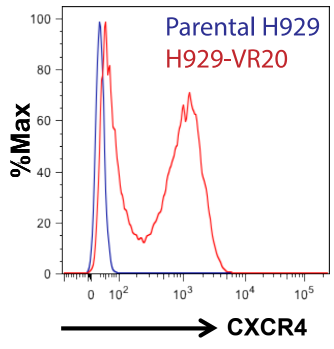


**i**

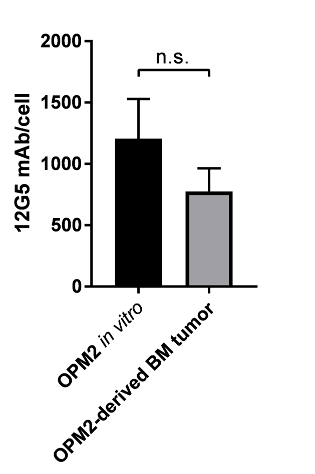


**Supplementary Fig. 3 (related to Fig. 3)**

**Supplementary Fig. 3 (related to Fig. 3). a, c, e,** Kinetics of orthothopic tumor growth (whole body tumour burden) after single dose at 3 mg/kg (arrows) of various m17-derived ADC configurations, p/sec = photos/second, Unconjugated m17 (mAb backbone for ADC 381) bearing active Fc-mediated effector function was included as a control. LP = linker-payload, Fc (+) = active Fc-mediated effector function, Fc (-) = reduced Fc-mediated effector function, SEM = standard error of the mean, (**a**) *P=0.016, (**c**) **P=0.004, (**e**) **P=0.002, all comparisons: two-way ANOVA with Sidak's multiple comparisons test. **b, d, f,** Kaplan-Meier analysis of median survival of mice in each treatment group, (**b**) **P=0.003, (**d**) *P=0.02, (**f**) **P=0.04. **a-f,** N=5 mice/group. **g,** Concentration-response curves showing relative sensitivity of MM-derived cell lines to SoC Velcade/bortezomib *in vitro*. H929-VR20 is derived from H929 cell line upon selection with 20 nM Velcade/bortezomib treatment *in vitro*. **h,** Expression of CXCR4 in the MM cell line H929 (parental H929) and in its subpopulation H929-VR20. Expression was measured using 12G5 anti-CXCR4 antibody by flow cytometry. **i,** Quantification of CXCR4 surface density (12G5 antibody binding) in the MM-derived cell line OPM2 (N=4 independent measurements) and in tumours isolated from bone marrow (BM) 36 days after i.v. OPM2 cell inoculation in tail vein of NSG mice (N=3 mice). Error bars = standard error of the mean, n.s. = non-significant (unpaired t test).

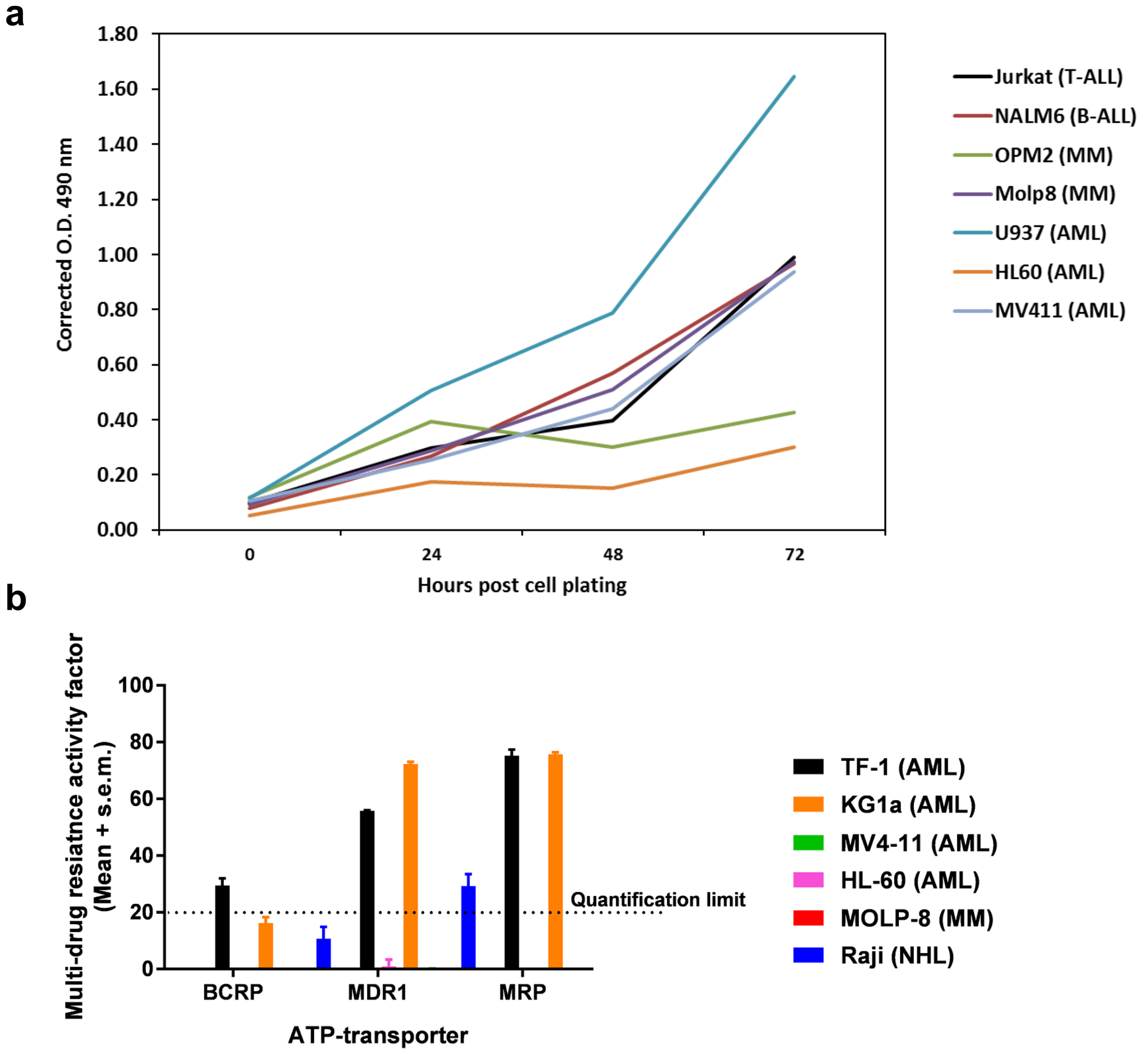


**Supplementary Fig. 4. AML cell lines used in the *in vitro* ADC cytotoxicity screen do not show different *in vitro* proliferation rates or increased activity of ATP transporters associated with multi-drug resistance, as compared to cell lines derived from other cancers. a,** Growth kinetics of human cell lines determined by XTT assay upon plating of 10,000 cells/well in 96-well plates. XTT was added to cells during the last 4 hours of incubation for each time point. O.D. = optical density. **b,** Activity of ATP transporters associated with multi-drug resistance. The AML cell lines KG1a and TF-1 were used as positive controls in this assay. BCRP = breast cancer resistance protein, MDR1 = multidrug resistance protein 1 (P-glycoprotein 1), MRP = Multidrug Related Protein, s.e.m. = standard error of the mean.

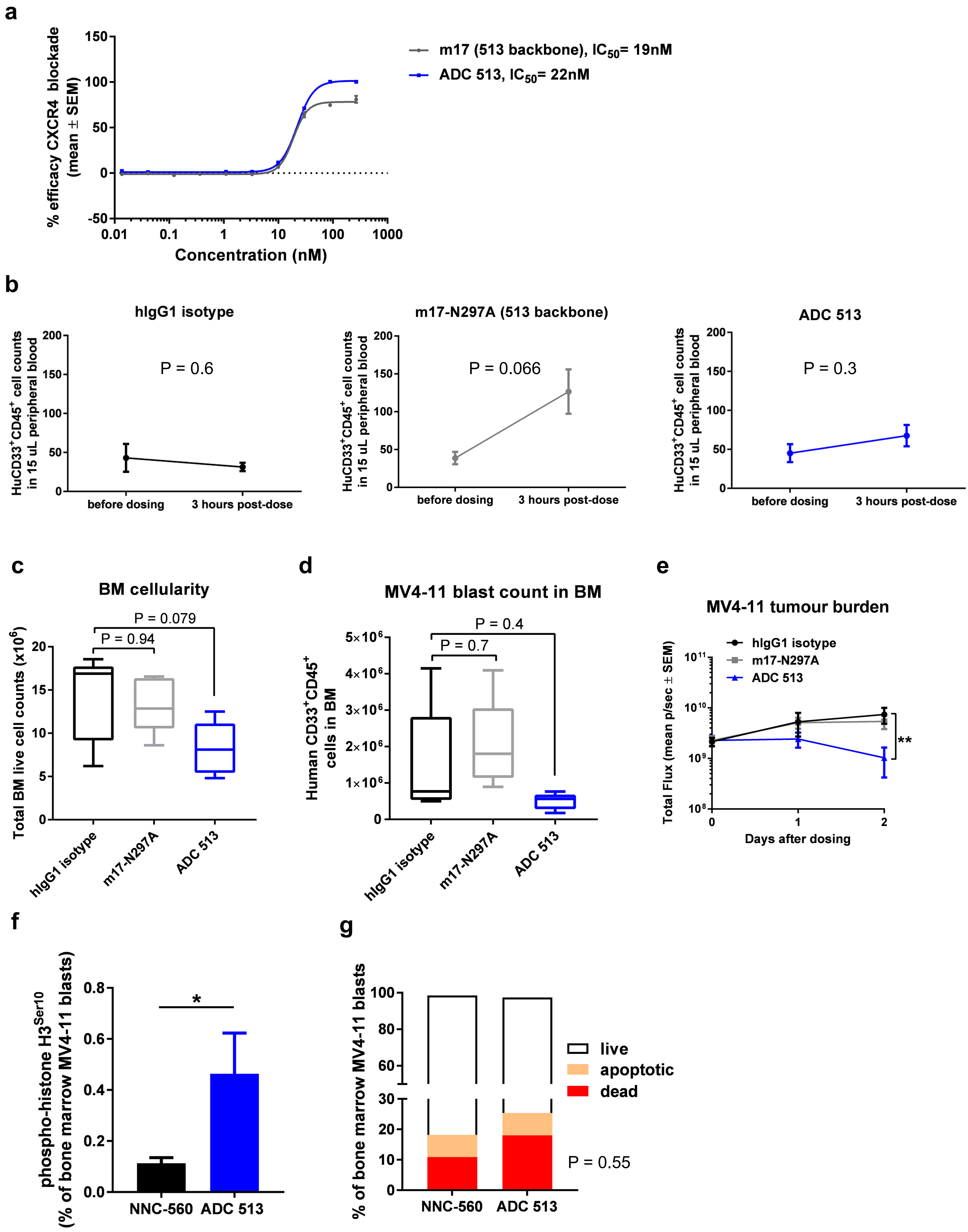


Supplementary Fig. 5

**Supplementary Fig. 5. MoA of anti-CXCR4 ADC-mediated cytotoxicity *in vivo*. a**, Bioassay of CXCR4 blockade by ADC 513 and respective unconjugated antibody in the presence of $EC_{80}$ forskolin and $EC_{80}$ CXCL12, SEM = standard error of the mean. **b-d,** Peripheral blood (**b**) and femur bone marrow (**c-d**) and were harvested 3 hours post-dose from same MV4-11 xenografts, N=5 mice/group. **b-g,** all compounds were single dosed at 3 mg/kg. **b,** Tumour leucocytosis assay in MV4-11 orthotopic xenografts with similar tumour burden as that of efficacy experiment (see Fig. 3g), N=5 mice/group, error bars = standard error of the mean, P values calculated using a paired t-test. **c,** Bone marrow (BM) cellularity. **d,** MV4-11 blast count in bone marrow (BM) of same mice as in b. **c-d**, P value calculated with ANOVA with Tukey's multiple comparisons test. **e**, Kinetics of MV4-11 orthotopic tumour burden, mice were dosed in parallel cohort (and with same tumour burden) as mice from b-d, **P = 0.004, two-way ANOVA with Dunnett's multiple comparisons test. **a-e,** m17-N297A corresponds to unconjugated mAb backbone of ADC 513. **f-g,** Mice with high MV4-11 orthotopic tumour burden were dosed with ADC 513 (N=3) or control ADC (N=4) and sacrificed 1.5 hours later for flow cytometric analysis of AML blasts in bone marrow. **f,** Phosphorylation levels of histone-$H3^{Ser10}$ in MV4-11 blasts, error bars = standard error of the mean, *P < 0.05 (unpaired t test). **g**, Proportion of viable, apoptotic and dead MV4-11 blasts by AnnexinV/propidium iodide staining (same samples as in f), P value (for proportion of dead cells) calculated by ANOVA with Sidak's multiple comparisons test.

**Supplementary Table 4.** ***In vitro*** **cytotoxicity screening of h17 variants-derived ADCs on cell lines from haematological cancers.** See also Table 1 for ADC configuration and properties and Supplementary Table 2 for CXCR4 surface density. Data for the most efficacious ADC for each cell line is highlighted in bold.

| | **Binding on $CXCR4^{+}$ cells** | **$IC_{50}$ (nM)** | | | | |
|---|---|---|---|---|---|---|
| | | **Ramos (NHL)** | **Daudi (NHL)** | **U266 (MM)** | **OPM2 (MM)** | **MOLP-8 (MM)** |
| **NNC-217** | **N/A** | >267 | >267 | 69.533 | >267 | >267 |
| **Aur0101** | **N/A** | 0.082 | 0.198 | 0.108 | 0.137 | 0.306 |
| **555** | **Medium** | 0.206 | 8.053 | 0.117 | 0.400 | 0.879 |
| **556** | **Low** | 0.176 | **3.775** | 0.118 | 0.756 | 1.027 |
| **554** | **High** | **0.111** | 10.320 | **0.013** | **0.097** | **0.749** |
| **553** | **Medium** | 0.229 | 10.687 | 0.016 | 0.360 | 0.983 |

a

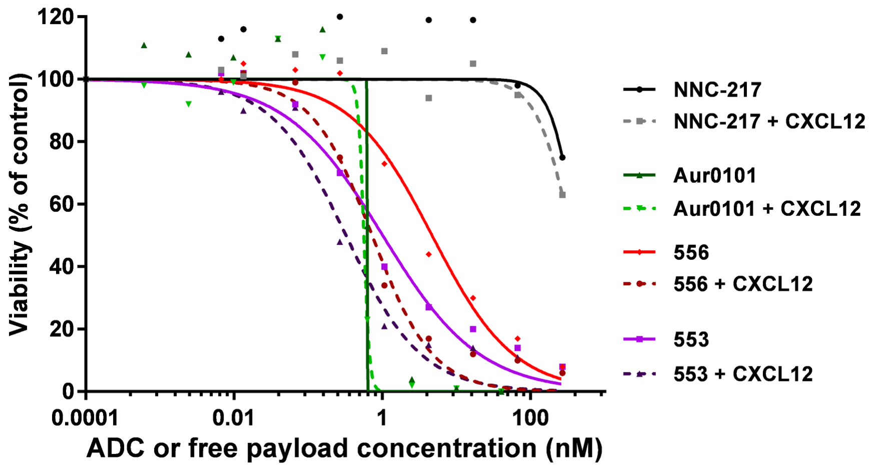


b

| | $IC_{50}$ (nM) |
|---|---|
| NNC-217 | >267 |
| NNC-217 + CXCL12 | >267 |
| Aur0101 | 0.625 |
| Aur0101+ CXCL12 | 0.562 |
| 556 | 4.677 |
| 556 + CXCL12 | 0.726 |
| 553 | 1.011 |
| 553 + CXCL12 | 0.323 |

c

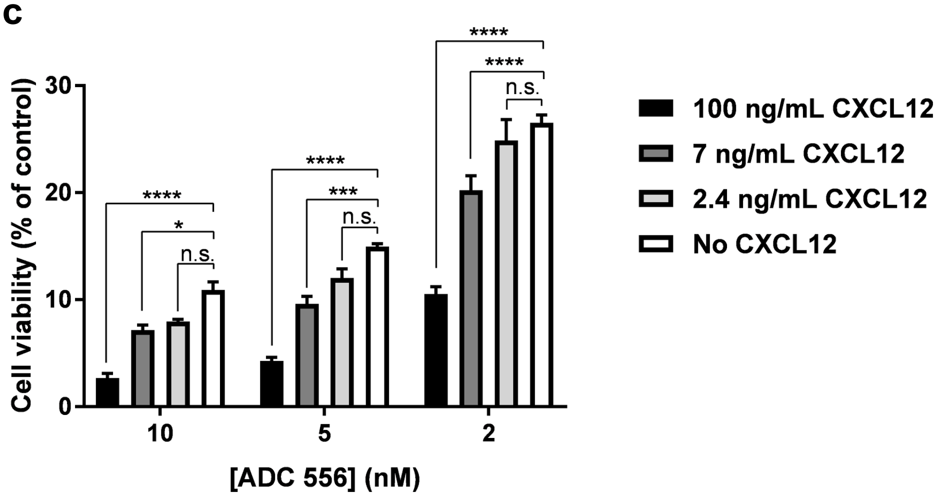


d

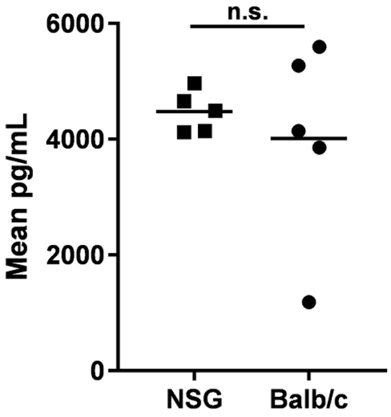


**Supplementary Fig. 6 (related to Fig.4). CXCL12 enhances anti-tumour activity of anti-CXCR4 ADCs in CXCR4$^{Low}$ tumour cells. a-b,** Effect of 100 ng/mL CXCL12 on the cytotoxicity (dose-response) of medium (ADC 553) and low (ADC 556) cell binding ADCs on the CXCR4$^{Low}$ MM-derived cell line OPM2. Data points are mean from triplicates of one experiment, with similar results observed in 2 other independent experiments. **c,** Effects of pathophysiologically relevant CXCL12 levels on the cytotoxicity of low cell binding ADC 556 on the CXCR4$^{Low}$ MM-derived cell line OPM2. High concentration (100 ng/mL) of CXCL12 was used as a positive control (based on results of experiments illustrated in panels a-b), 7 ng/mL is the average CXCL12 concentration reported in bone marrow samples of MM patients and 2.4 ng/mL is the average CXCL12 concentration reported in peripheral blood of same patients. Error bars = standard error of the mean, n.s. = non-significant, *P=0.01, ***P=0.0006, ****P<0.0001 (two-way ANOVA with Dunnett's multiple comparisons test). **d,** ELISA determination of CXCL12 levels in the serum of immuno-deficient (NSG) and immuno-competent (Balb/c) tumour-naïve mice. Each data point represents result from one animal, n.s. = non-significant (unpaired t test).

**Supplementary Table 5.** ***In vitro*** **cytotoxicity and specificity of h17-NV.TS- and h17-NA- derived, AmPEG6C2-Aur0131-conjugated (DAR4) ADCs.** See also Table 1 for ADC configuration and properties.

| | Binding on CXCR4+ cells | $IC_{50}$ (nM) | |
|---|---|---|---|
| | | Jurkat (CXCR4++++) | CHO (CXCR4-) |
| **NNC-715** | N/A | >267 | >267 |
| **NNC-560** | N/A | >267 | >267 |
| **Aur0131 (Ome)** | N/A | 1.675 | 3.997 |
| **711** | Medium | 0.245 | >267 |
| **712** | Medium | 0.262 | >267 |
| **713** | Low | 0.294 | >267 |
| **714** | Low | 0.282 | >267 |

a

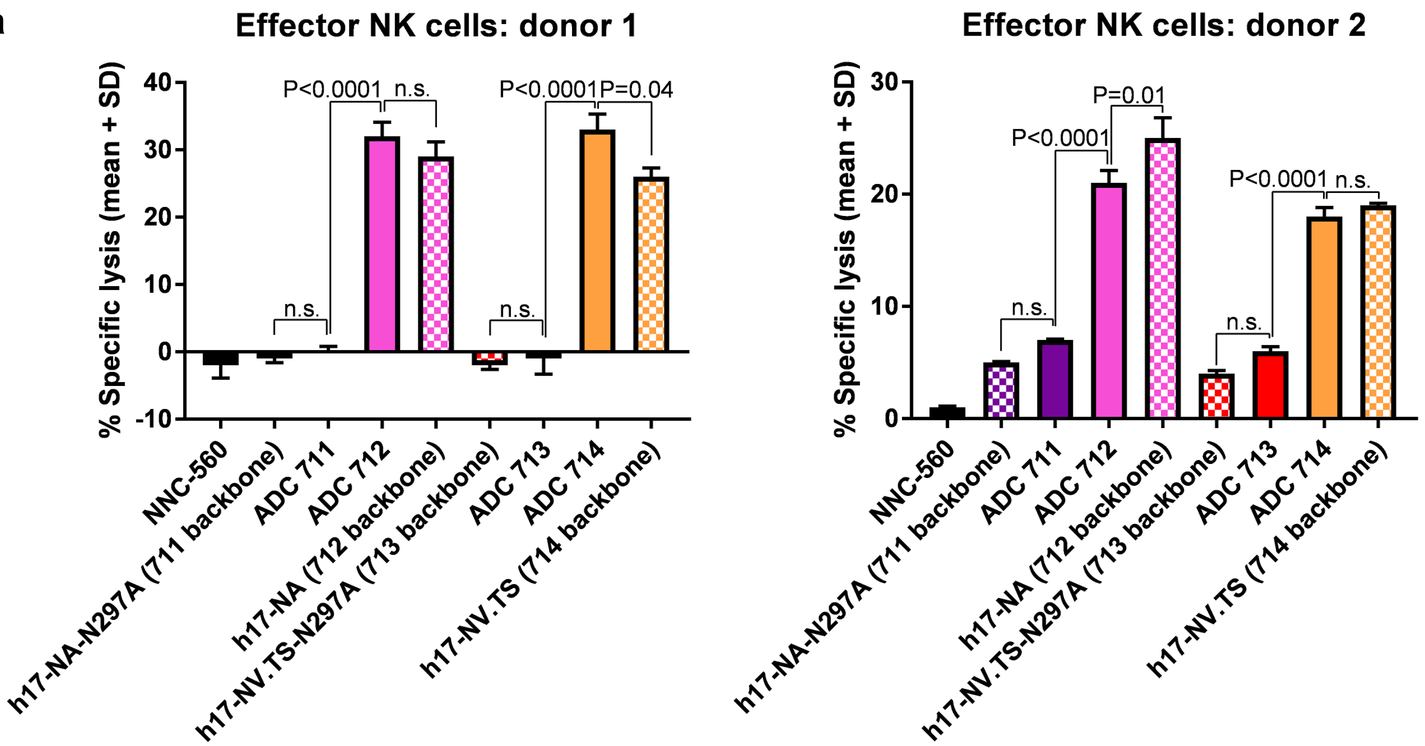


b

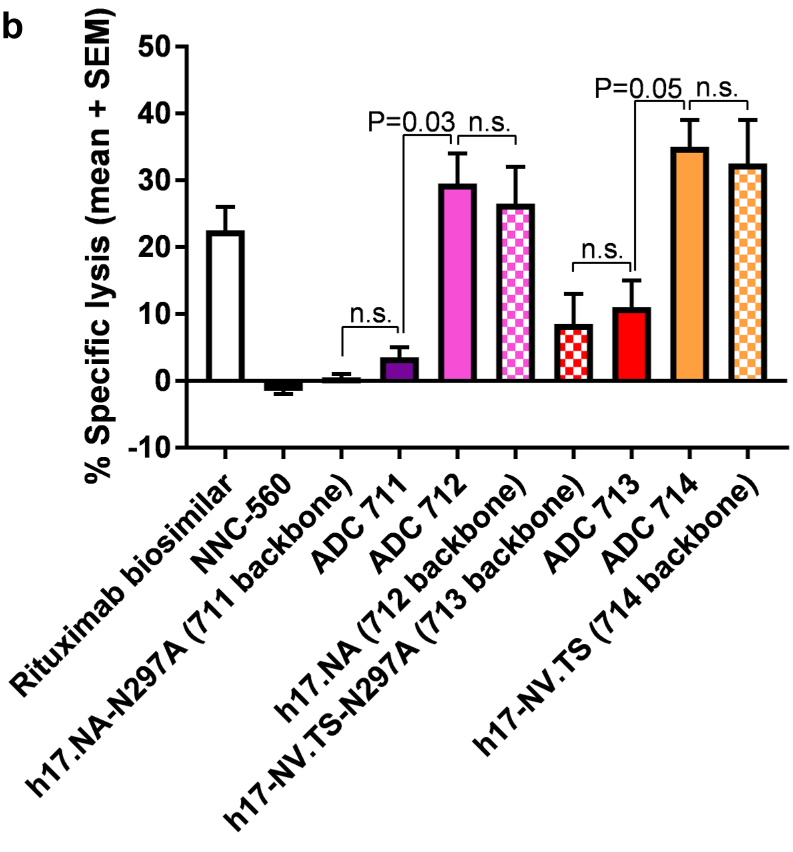


**Supplementary Fig. 7 (related to Fig.5). Fc-mediated effector function in humanized ADCs is equivalent to that of respective unconjugated antibodies. a**, Result of *in vitro* ADCC assay on MOLT-4 cell line (T-ALL), using 5 µg/mL h17-derived ADCs, or their respective unconjugated antibody and human NK cells as effectors. Effector-to-target cells ratio = 1:10. Data shown are mean + standard deviation (SD) of duplicates per condition, with 2 independent experiments using different NK cells donors. One-way ANOVA with Tukey's multiple comparisons test used for statistical analysis, n.s. = non significant). **b**, Result of *in vitro* CDC assay on Daudi cell line (NHL), using 7 µg/mL h17-derived ADCs, or their respective unconjugated antibody backbones and 2.5% human serum. A rituximab biosimilar was included as a positive control. Data shown are mean of 2 independent experiments. One-way ANOVA with Tukey's multiple comparisons test used for statistical analysis, n.s. = non significant, SEM= standard error of the mean.

**Supplementary Table 6. Pharmacokinetic parameters of h17-NV.TS- and h17-NA- derived, AmPEG6C2-Aur0131-conjugated (DAR4) ADCs in MOLP-8 xenografts and HuCXCR4KI mice.** Mice were single dosed with indicated ADCs through the tail vein. MOLP-8 tumour burden is similar to that of efficacy studies at day of dosing. See also Table 1 for ADC configuration and properties.

| Mouse Strain/Model | ADC | Cell binding | Fc | Dose (mg/kg) | Assay | Cmax (mg/mL) | Tmax (hr) | $AUC_{0-x}$ (hr*mg/mL) | AUC interval (hr) | T1/2 (hr) |
|---|---|---|---|---|---|---|---|---|---|---|
| HuCXCR4KI | 711 | Medium | (-) | 3.0 | Conjugated mAb | 23.1 | 6 | 1208 | 0-504 | 48 |
| HuCXCR4KI | 711 | Medium | (-) | 3.0 | Total mAb | 23.2 | 6 | 1243 | 0-504 | 24 |
| HuCXCR4KI | 713 | Low | (-) | 3.0 | Conjugated mAb | 21.5 | 6 | 1564 | 0-504 | 48 |
| HuCXCR4KI | 713 | Low | (-) | 3.0 | Total antibody | 21.5 | 6 | 1658 | 0-504 | 35 |
| HuCXCR4KI | 711 | Medium | (-) | 10.0 | Conjugated mAb | 281.0 | 0.5 | 10600 | 0-168 | 73 |
| HuCXCR4KI | 711 | Medium | (-) | 10.0 | Total antibody | 292.0 | 0.5 | 11000 | 0-168 | 71 |
| HuCXCR4KI | 713 | Low | (-) | 10.0 | Conjugated mAb | 153.0 | 0.5 | 8090 | 0-168 | 78 |
| HuCXCR4KI | 713 | Low | (-) | 10.0 | Total antibody | 169.0 | 0.5 | 9200 | 0-168 | 62 |
| MOLP-8 Xenograft | 711 | Medium | (-) | 3.0 | Conjugated mAb | 40.5 | 6 | 10575 | 0-504 | 233 |
| MOLP-8 Xenograft | 711 | Medium | (-) | 3.0 | Total antibody | 40.6 | 6 | 11044 | 0-504 | 251 |
| MOLP-8 Xenograft | 712 | Medium | (+) | 3.0 | Conjugated mAb | 42.5 | 6 | 3828 | 0-504 | 39 |
| MOLP-8 Xenograft | 712 | Medium | (+) | 3.0 | Total antibody | 42.8 | 6 | 4074 | 0-504 | 31 |
| MOLP-8 Xenograft | 713 | Low | (-) | 3.0 | Conjugated mAb | 37.8 | 6 | 10230 | 0-504 | 321 |
| MOLP-8 Xenograft | 713 | Low | (-) | 3.0 | Total antibody | 37.3 | 6 | 10510 | 0-504 | 340 |
| MOLP-8 Xenograft | 714 | Low | (+) | 3.0 | Conjugated mAb | 33.3 | 6 | 2189 | 0-504 | 42 |
| MOLP-8 Xenograft | 714 | Low | (+) | 3.0 | Total antibody | 30.5 | 6 | 2161 | 0-504 | 30 |
| MOLP-8 Xenograft | 711 | Medium | (-) | 0.03 | Conjugated mAb | 0.092 | 6 | 18 | 0-336 | 107 |
| MOLP-8 Xenograft | 711 | Medium | (-) | 0.03 | Total antibody | 0.090 | 6 | 14 | 0-336 | 186 |
| MOLP-8 Xenograft | 711 | Medium | (-) | 0.1 | Conjugated mAb | 0.41 | 6 | 88 | 0-504 | 284 |
| MOLP-8 Xenograft | 711 | Medium | (-) | 0.1 | Total antibody | 0.43 | 6 | 81 | 0-504 | 268 |
| MOLP-8 Xenograft | 711 | Medium | (-) | 0.3 | Conjugated mAb | 1.33 | 6 | 265 | 0-504 | 335 |
| MOLP-8 Xenograft | 711 | Medium | (-) | 0.3 | Total antibody | 1.51 | 6 | 270 | 0-504 | 278 |
| MOLP-8 Xenograft | 713 | Low | (-) | 0.05 | Conjugated mAb | 0.50 | 6 | 64 | 0-336 | 209 |
| MOLP-8 Xenograft | 713 | Low | (-) | 0.05 | Total antibody | 0.46 | 6 | 66 | 0-336 | 186 |
| MOLP-8 Xenograft | 713 | Low | (-) | 0.15 | Conjugated mAb | 1.82 | 6 | 329 | 0-504 | 243 |
| MOLP-8 Xenograft | 713 | Low | (-) | 0.15 | Total antibody | 1.58 | 6 | 291 | 0-504 | 267 |
| MOLP-8 Xenograft | 713 | Low | (-) | 0.5 | Conjugated mAb | 6.82 | 6 | 1272 | 0-504 | 237 |
| MOLP-8 Xenograft | 713 | Low | (-) | 0.5 | Total antibody | 5.83 | 6 | 1075 | 0-504 | 247 |

Hr = hours. AUC = Area under the concentration curve. Cmax = peak serum concentration. Tmax = time taken to reach the maximum concentration. T1/2 = time taken for Cmax to drop in half. (-) = Reduced Fc-mediated effector function. (+) = Active Fc-mediated effector function.

**Supplementary Table 7. Tolerability of ADCs 711 and 713 in HuCXCR4KI mice.** See also Table 1 for ADC configuration and properties and Fig.6a for dosing schedule.

| ADC and mouse genotype | Dose (mg/kg/dose) | Tolerability after 1st dose | | Tolerability after 2nd dose | | Tolerability after 3rd dose | |
|---|---|---|---|---|---|---|---|
| | | males | females | males | females | males | females |
| 711 (Medium cell binding) in HuCXCR4KI | 10.0 | 3/6 (50%) | 0/6 (0%) | | | | |
| | 8.0 | | | | | | |
| | 6.0 | 0/5 (0%) | 4/5 (80%) | | 4/5 (80%) | | |
| | 4.5 | 4/5 (80%) | | | | | |
| | 3.0 | 9/10 (90%)* | 6/6 (100%) | 9/10 (90%) | 6/6 (100%) | 9/10 (90%) | 6/6 (100%) |
| 713 (Low cell binding) in HuCXCR4KI | 10.0 | 6/6 (100%) | 4/6 (67%) | 4/6 (67%) | 3/6 (50%) | | |
| | 8.0 | 4/5 (80%) | | 4/5 (80%) | | | |
| | 6.0 | 5/5 (100%) | 4/5 (80%) | 5/5 (100%) | 2/5 (40%) | 5/5 (100%) | |
| | 4.5 | 5/5 (100%) | 5/5 (100%) | 5/5 (100%) | 5/5 (100%) | 5/5 (100%) | 5/5 (100%) |
| | 3.0 | 10/10 (100%) | 6/6 (100%) | 10/10 (100%) | 6/6 (100%) | 10/10 (100%) | 6/6 (100%) |
| 711 (Medium cell binding) in WT | 10.0 | 6/6 (100%) | 6/6 (100%) | 6/6 (100%) | 6/6 (100%) | 6/6 (100%) | 6/6 (100%) |

*1/10 males died after first dose. We presume the death is likely not test article-related because all other mice of both genders tolerated the multiple dose study.

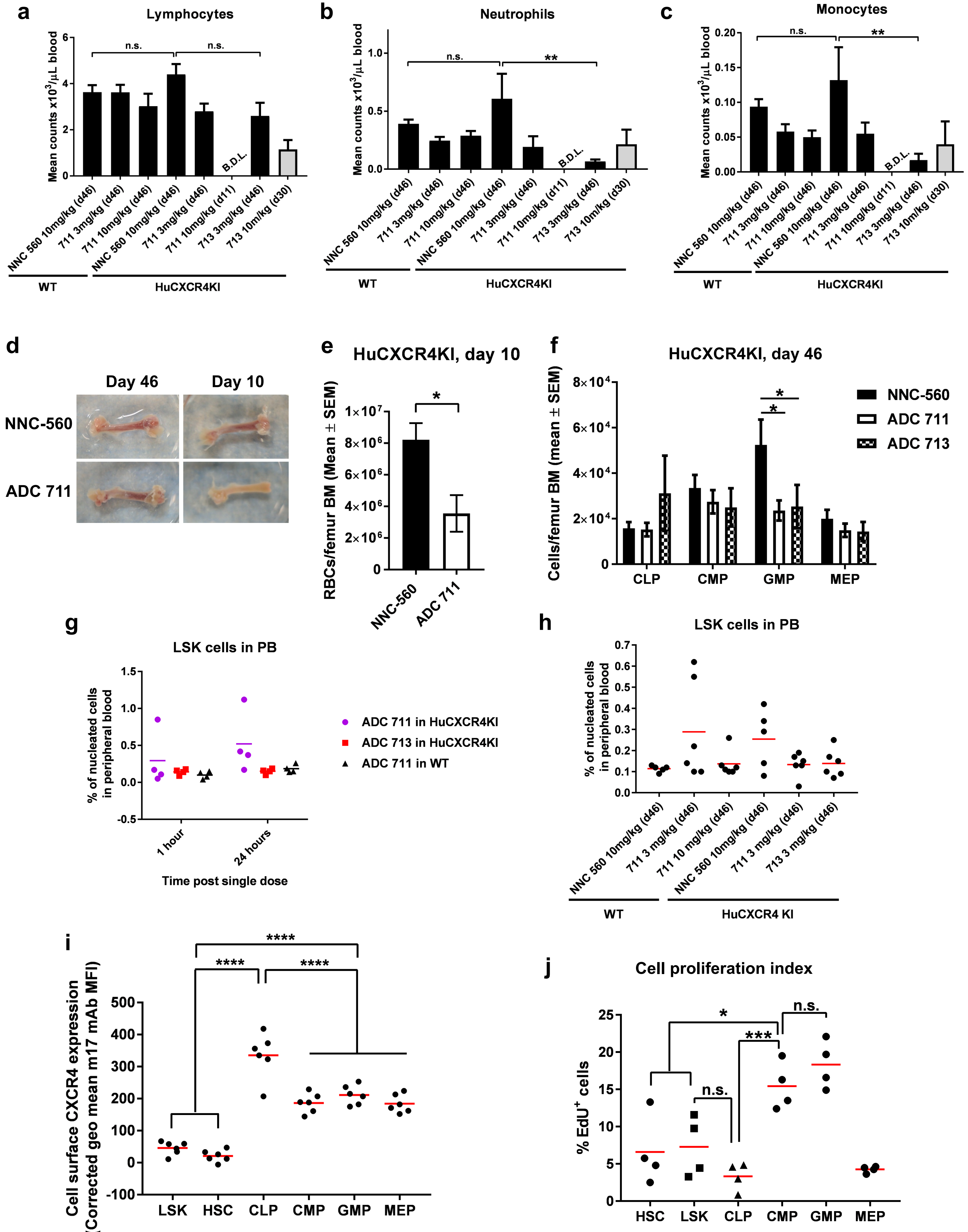


Supplementary Fig. 8 (related to Fig. 6)

**Supplementary Fig. 8 (related to Fig. 6). a-c,** Hematology data from tolerability study. The necropsy day is indicated in parenthesis, in x-axis. Black bars are mean from scheduled necropsy on day 46 (N=6/group), grey bars are mean from unscheduled necropsies on days 11 and 30 (N=3/group). B.D.L. = below detection limit, error bars = standard error of the mean. One-way ANOVA with Tukey's multiple comparisons test for data collected at day 46, n.s. = non-significant (**b**) **P=0.004 (**c**) **P=0.005. **d-e,** Red blood cells in femur bone marrow decrease after single 3 mg/kg dose of ADC711 (day 10), but recovery is observed at day 46, in spite of HuCXCR4KI receiving two additional doses (day 46). See also Figs. 6a and 6f. **e,** Red blood cell (RBCs) quantification in femur bone marrow at day 10, SEM = standard error of the mean, *P=0.02, unpaired t test. **f**, Enumeration of specific hematopoietic progenitor populations in femur bone marrow (BM) of HuCXCR4KI mice by flow cytometry at day 46 of the tolerability study (dose level 3 mg/kg/dose), N=5/group, SEM = standard error of the mean, *P <0.03, two-way ANOVA with Dunnett's multiple comparisons test. **g-j,** Each symbol represents data from an individual mouse. **g-h,** Frequency of hematopoietic stem cells and progenitors - $Lin^{-}Sca1^{+}c\text{-}kit^{+}$ (LSK) - measured by flow cytometry in peripheral blood (PB) after either single 3 mg/kg dose (**g**) or repeat dosing at necropsy day 46 (**h**). No statistically significant differences were found in either experiment by two-way ANOVA with Tukey's multiple comparisons test (**g**) and one-way ANOVA with Dunnett's multiple comparisons test. **g-h,** HSC = hematopoietic stem cells, CLP = common lymphoid progenitor, CMP = common myeloid progenitor, GMP = granulocyte-monocyte progenitor, MEP = megakaryocyte-erythroid progenitor. **i,** Cell surface CXCR4 expression levels in hematopoietic progenitor populations from femur bone marrow of HuCXCR4KI mice in homeostatic conditions, measured with m17 mAb directly conjugated to AlexaFluor647, ****P<0.0001 (one-way ANOVA with Tukey's multiple comparisons test). **j,** Cell proliferation index of hematopoietic progenitors in femur bone marrow of HuCXCR4KI mice in homeostatic conditions (non-treated), n.s. = non-significant, *P<0.02, ***P=0.0008 (one-way ANOVA with Tukey's multiple comparisons test).

a

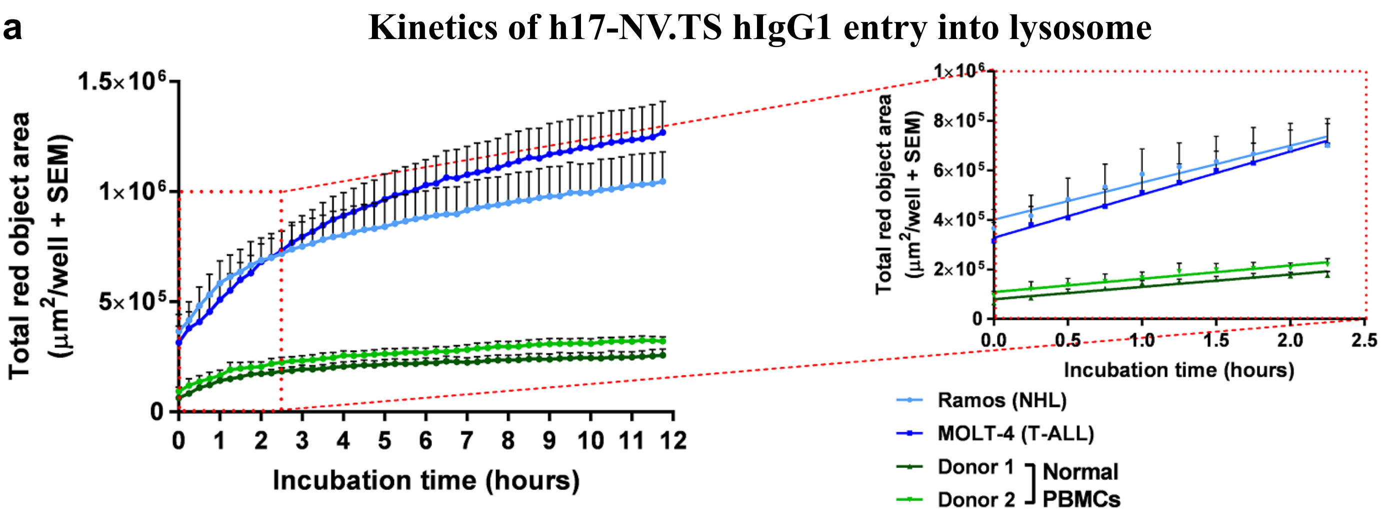


b

| h17-NV.TS internalization rate during first 2.5 hours incubation (Best-fit slope ± SE) | | | |
|---|---|---|---|
| Ramos | MOLT-4 | PBMC Donor 1 | PBMC Donor 2 |
| 149,379 ± 11,684 | 174,310 ± 4,467 | 49,933 ± 4,820 | 53,554 ± 4,260 |

c

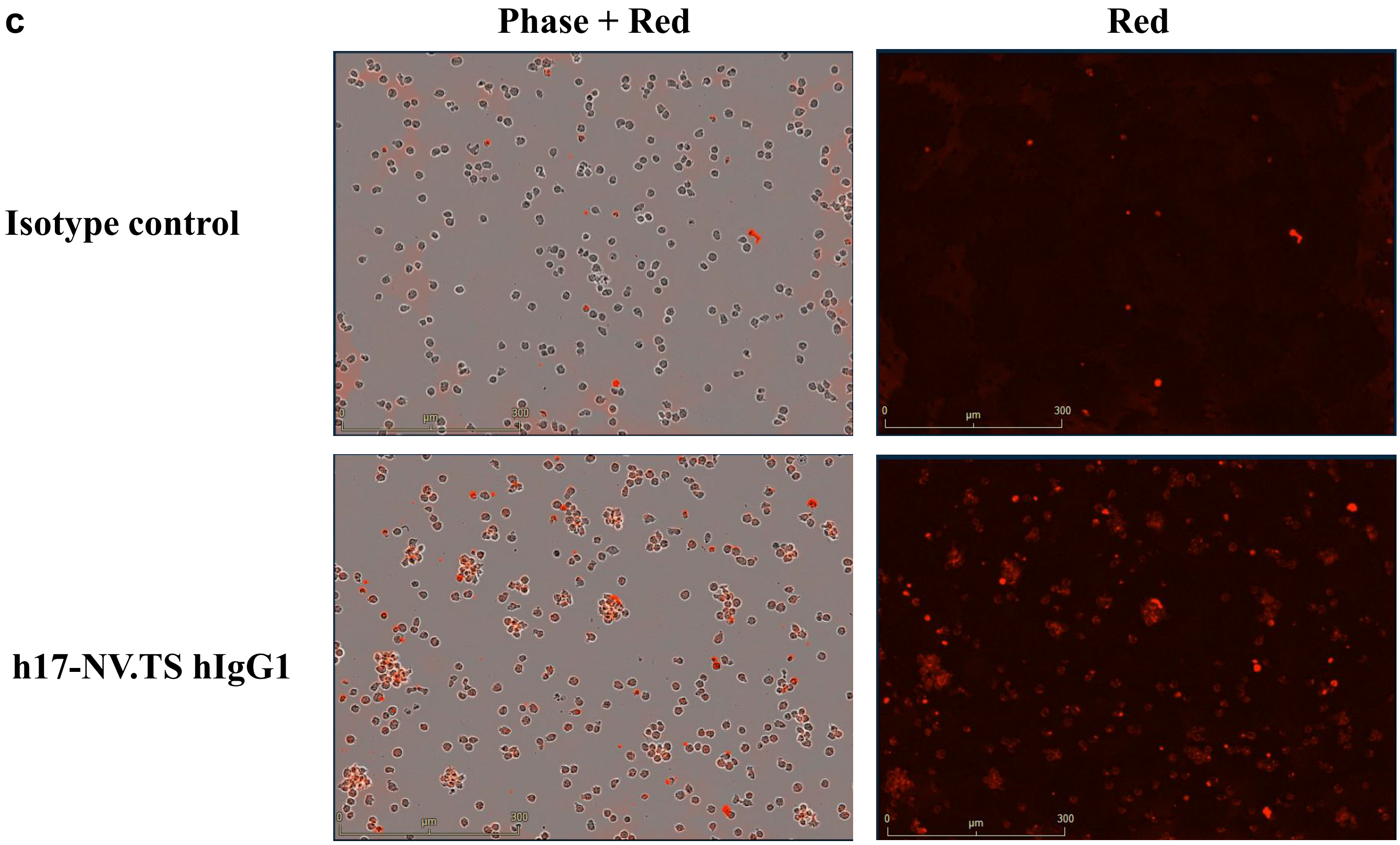


d

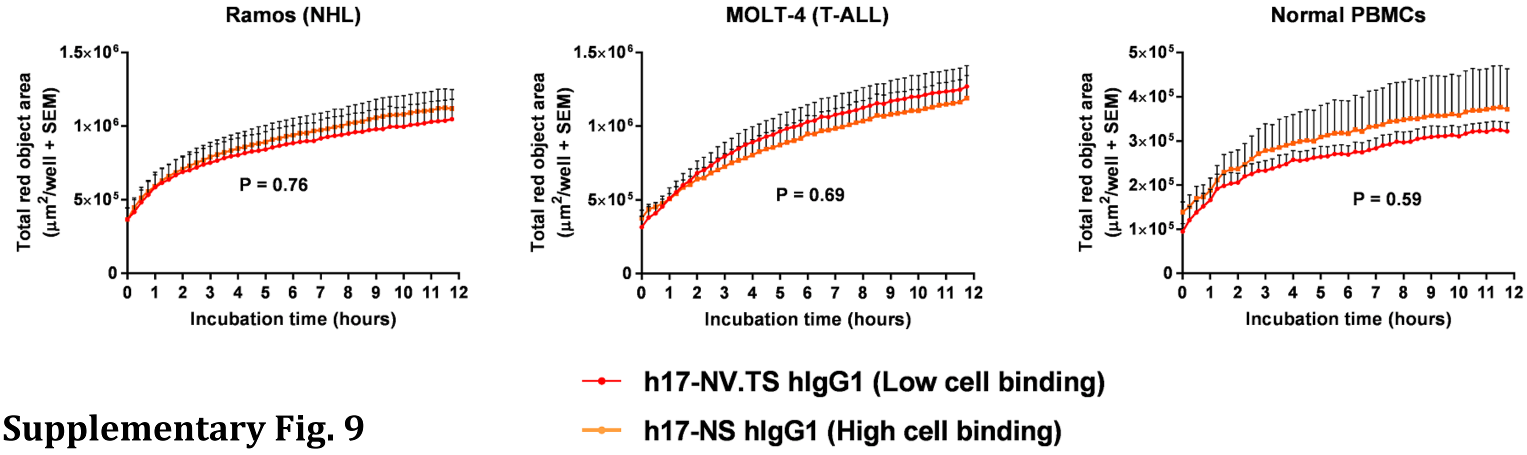


Supplementary Fig. 9

**Supplementary Fig. 9. Kinetics of internalization of CXCR4:antibody complexes is faster in human cancer cells than in normal PBMCs. a, d,** Recorded fluorescent signal of pH-sensitive fluorophore-labeled antibodies upon addition to cells (3 µg/mL antibody) and start of incubation at 37 °C, SEM = standard error of the mean (from triplicate wells). **a,** Internalization rates of CXCR4:h17-NV.TS antibody complexes in cancer cells and normal PBMCs. Linear regression analysis was applied to the data from up to 2.5 hours incubation, at which time point fluorescent probe signal in normal PBMCs reaches plateau. **b,** Internalization rates: linear regression of variation in total red object area over the first 2.5 hours of incubation time (from a, right panel), SE = standard error. **c,** Micrographs of MOLT-4 cells at 6 hours post incubation with 3 µg/mL h17-NV.TS antibody show high intracellular (red) signal resultant from fluorescent probe exposure to low pH in lysosome. No specific signal is detected with isotype control at the same concentration and time point. **d,** Internalization rates of high (h17-NS) and low (h17-NV.TS) cell binding anti-CXCR4 antibody variants in cancer cell lines and normal PBMCs. P values calculated using two-way ANOVA with Sidak's multiple comparisons test.
